# Alternative Splice Forms Influence Functions of Whirlin in Mechanosensory Hair Cell Stereocilia

**DOI:** 10.1016/j.celrep.2016.03.081

**Published:** 2016-04-21

**Authors:** Seham Ebrahim, Neil J. Ingham, Morag A. Lewis, Michael J.C. Rogers, Runjia Cui, Bechara Kachar, Johanna C. Pass, Karen P. Steel

**Affiliations:** 1 Wolfson Centre for Age-Related Diseases, King’s College London, Guy’s Campus, London SE1 1UL, UK; 2 Wellcome Trust Sanger Institute, Hinxton, Cambridge CB10 1SA, UK; 3 MRC Institute of Hearing Research, Nottingham NG7 2RD, UK; 4 National Institute on Deafness and Other Communications Disorders, NIH, Bethesda, MD 20892, USA

## Abstract

*WHRN* (*DFNB31*) mutations cause diverse hearing disorders: profound deafness (DFNB31) or variable hearing loss in Usher syndrome type II. The known role of WHRN in stereocilia elongation does not explain these different pathophysiologies. Using spontaneous and targeted *Whrn* mutants, we show that the major long (WHRN-L) and short (WHRN-S) isoforms of WHRN have distinct localizations within stereocilia and also across hair cell types. Lack of both isoforms causes abnormally short stereocilia and profound deafness and vestibular dysfunction. WHRN-S expression, however, is sufficient to maintain stereocilia bundle morphology and function in a subset of hair cells, resulting in some auditory response and no overt vestibular dysfunction. WHRN-S interacts with EPS8, and both are required at stereocilia tips for normal length regulation. WHRN-L localizes midway along the shorter stereocilia, at the level of inter-stereociliary links. We propose that differential isoform expression underlies the variable auditory and vestibular phenotypes associated with *WHRN* mutations.

## Highlights

• Major WHRN isoforms WHRN-S and WHRN-L have distinct localizations within stereocilia
• Lack of WHRN-S and WHRN-L causes short stereocilia bundles and profound deafness
• In absence of WHRN-L, WHRN-S can preserve stereocilia length in certain hair cells
• Differential isoform expression underlies distinct phenotypes of known *Whrn* mutations

## Graphical Abstract

**Figure:**
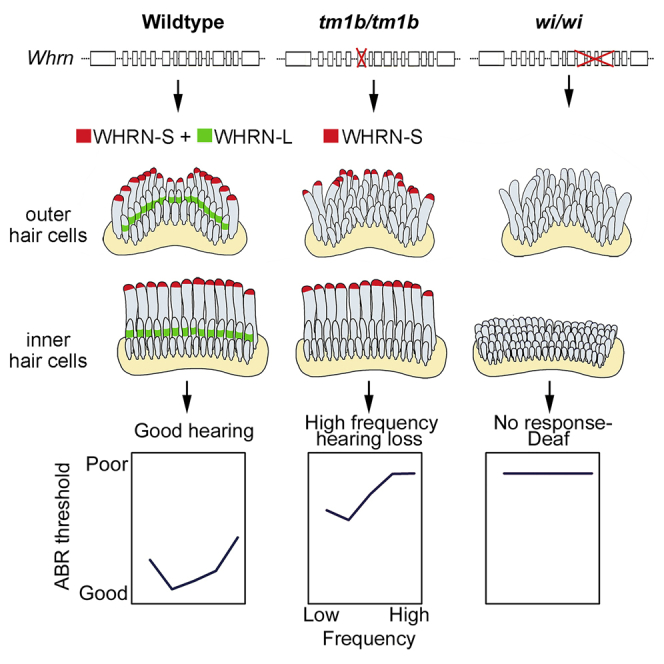

## Teaser

Ebrahim et al. show that two major isoforms of the *WHRN* gene have distinct localizations and functions within and across mechanosensory hair cells in the inner ear, and isoform-specific mutations are the likely cause of different auditory pathophysiologies associated with *WHRN* in mouse and humans.

## Introduction

Inner ear sensory hair cells (HCs) transduce sound and head motion to electrical impulses via their mechanosensory hair bundles. Each hair bundle comprises dozens of specialized actin-filled protrusions, called stereocilia, organized in rows of graded heights. Stereocilia heights and organization have important effects on the HC’s operating range, sensitivity, and frequency selectivity (Aranyosi and Freeman, 2004), and a number of proteins involved in regulating stereocilia morphology are essential for normal hearing (Dror and Avraham, 2009).

The PDZ domain-containing protein whirlin (WHRN) has been shown to localize to the tips of stereocilia, where it is involved in length-regulation (Delprat et al., 2005, Holme et al., 2002, Manor et al., 2011), and also to the stereocilia base, where it is thought to play a role in the bundle organization during development (Delprat et al., 2005). *Whrn* consists of 13 exons with two major splice variants: a long isoform (referred to here as WHRN-L) encoded by exons 1–13, composed of two PDZ domains at the N terminus followed by a proline-rich domain and a third PDZ at the C terminus; and a short form (WHRN-S), encoded by exons 6–13, which lacks PDZ1 and PDZ2 of the N terminus (Mburu et al., 2003). Mutations in *DFNB31* (encoding WHRN) cause profound non-syndromic deafness, DFNB31 (Mburu et al., 2003, Mustapha et al., 2002, Tlili et al., 2005). *DFNB31* has also been associated with Usher syndrome II, involving retinal degeneration and variable hearing loss (Aller et al., 2010, Audo et al., 2011, Ebermann et al., 2007). The underlying molecular pathogenic mechanisms are not known.

We addressed this outstanding question in the current study using the *Whrn*^wi/wi^ mouse, in which both major WHRN isoforms are ablated (Mburu et al., 2003), and a mouse mutant, *Whrn*^tm1b(KOMP)Wtsi^ (referred to here as *Whrn*^tm1b^), which expresses WHRN-S but not WHRN-L. While the *Whrn*^wi/wi^ mutant is profoundly deaf and exhibits circling and headbobbing behavior, we found that the *Whrn*^tm1b^ mutant shows only moderate to severe hearing loss, suggesting that WHRN-S is sufficient to prevent complete loss of auditory function as well as vestibular dysfunction. We show that normal stereocilia are maintained in cochlear inner hair cells (IHCs) of the *Whrn*^tm1b^ mutant, but outer hair cell (OHC) stereocilia morphology, organization, and function are affected. Similarly, while a subset of vestibular HCs in the *Whrn*^*t*m1b^ mutant have abnormally short stereocilia, the remainder have stereocilia bundles with close-to-normal morphology. We use immunofluorescence with super-resolution, structured illumination microscopy (SIM) to determine the spatiotemporal localization of both WHRN isoforms along stereocilia. We show that WHRN-S localizes to the tips of stereocilia of IHCs from birth to adulthood, colocalizes with the actin regulatory protein EPS8, and is required for normal stereocilia length regulation. We propose that the localization of WHRN-L along the stereocilia shaft coincides with inter-stereociliary links, such as lateral links or horizontal top connectors, and its absence leads to disorganized bundles. Thus, WHRN isoforms are expressed differentially across HC types and within stereocilia, where they play distinct roles in organization and elongation.

## Results

### *Whrn*^wi/wi^ and *Whrn*^tm1b/tm1b^ Mice Express Different WHRN Isoforms

**Figure 1:**
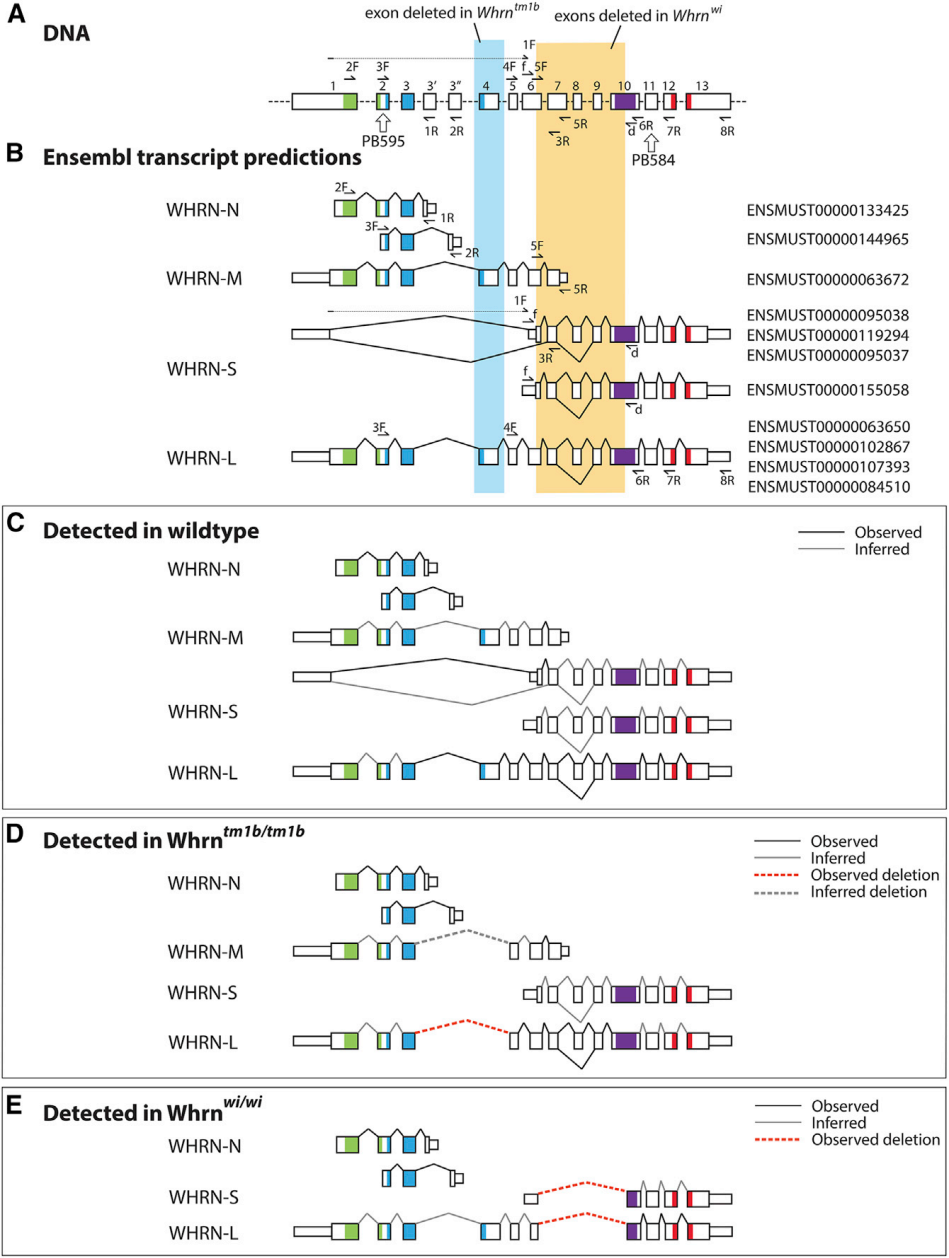
*Whrn* Isoform Expression (A) Diagram of *Whrn* exons showing deletions in *Whrn*^wi/wi^ (orange) and *Whrn*^tm1b/tm1b^ (blue), with regions coding for WHRN domains colored (green, PDZ1; blue, PDZ2; red, PDZ3; purple, proline-rich domain). Vertical arrows mark target region of antibodies PB584 and PB595. Horizontal arrows mark locations of primers. (B) Ensembl predictions of *Whrn* isoforms. Narrow boxes indicate UTRs and wider boxes protein-coding regions. Left: isoform names used in this paper. Right: Ensembl transcript IDs. ENSMUST00000155058 is classified as a retained intron transcript but was recently found in the inner ear (Mathur et al., 2015b) and results in a protein sequence identical to transcript ENSMUST00000119294, one of the WHRN-S isoforms, when translated. (GenBank accession numbers provided in Table S1). Primers used to detect each isoform are shown on the relevant isoform. (C–E) Transcripts detected in wild-type (C), *Whrn*^tm1b/tm1b^, (D) and *Whrn*^wi/wi^, and (E) inner ears. Black lines indicate splice junctions observed by sequencing. Grey lines indicate splice junctions inferred by presence of the isoform, but not observed by sequencing. See also Figure S1.

In this study, we analyzed the whirler (*Whrn*^wi/wi^) mouse (Mburu et al., 2003, Mogensen et al., 2007), which has a spontaneous deletion encompassing the majority of exons 6–10 of the *Whrn* gene, resulting in the ablation of both major isoforms of WHRN, WHRN-L and WHRN-S (Figures 1A and 1B). We also used a mutant, *Whrn*^tm1b(KOMP)Wtsi^ (referred to from here as *Whrn*^tm1b^), in which exon 4 of the *Whrn* gene is deleted and a cassette including a β-galactosidase reporter (lacZ), is inserted into intron 3 (Figures S1A and S1B).

Ensembl predicts five main WHRN isoforms: two short N-terminal isoforms (here referred to collectively as WHRN-N), a midsize isoform (WHRN-M), and several splice variants of both the long isoform (WHRN-L) and the short C-terminal isoform (WHRN-S) (Figure 1B). We examined the transcripts present in inner ear tissue of each mutant by reverse transcriptase (RT) PCR followed by capillary sequencing (Figure 1B).

In the wild-type inner ear, we found both WHRN-N isoforms, WHRN-M, WHRN-L, and two variants of WHRN-S (Figure 1C). The two WHRN-N isoforms were also found in both *Whrn*^wi^ and *Whrn*^tm1b^ homozygotes (Figures 1D and 1E). We were unable to test for the presence of WHRN-M in *Whrn*^wi^ homozygotes because the only specific primer set fell in the deletion; if this isoform is transcribed, it is predicted to be truncated. We did confirm the presence of WHRN-M in *Whrn*^tm1b^ homozygotes but could not observe the lack of exon 4 in this isoform because exon 4 was not part of the sequence amplified by the specific primer set (Figure 1D).

WHRN-L was detected in both *Whrn*^wi^ and *Whrn*^tm1b^ homozygotes (Figures 1D and 1E), and the deletion in each mutant allele was observed by sequencing. In the *Whrn*^wi^ sequence, there is a clean break from exon 6 to exon 10, which introduces a frameshift. The *Whrn*^tm1b^ sequence lacks exon 4 but has a section of 115 bp between exon 3 and exon 5 that is an exact match to the region around the splice junction of the second exon of *En2*. It is likely that this is because the trapping cassette used in making the original tm1a allele includes the mouse *En2* splice acceptor (Skarnes et al., 2011). If either the *Whrn*^wi^ or the *Whrn*^tm1b^ WHRN-L transcripts are translated, the mutations are predicted to introduce a stop codon and result in a truncated protein.

WHRN-S was also detected in both *Whrn*^wi^ and *Whrn*^*t*m1b^ homozygotes (Figures 1D and 1E). The *Whrn*^tm1b^ product was identical to the wild-type product. The deletion in the *Whrn*^wi^ allele was clear, just as observed in the WHRN-L transcript. In the WHRN-S isoform, the *Whrn*^wi^ deletion starts in the 5′UTR and removes the start codon, so it is unlikely that any protein is produced from the mutant transcript.

### *Whrn*^wi/wi^ and *Whrn*^tm1b/tm1b^ Mice Show Distinct Cochlear Physiology, Vestibular Phenotypes, and Whrn Expression Pattern

Phenotypic differences between the two mutants were immediately apparent: *Whrn*^wi/wi^ mice are profoundly deaf and exhibit headbobbing and circling behavior characteristic of severe vestibular dysfunction, while *Whrn*^tm1b/tm1b^ mice intriguingly showed no overt vestibular abnormalities. We tested the contact righting reflex, which uses a combination of visual, vestibular, and somatosensory inputs to make postural adjustments after displacement, in adult mice. When *Whrn*^tm1b/tm1b^ mice were placed on their backs, they immediately turned over to rest on all four feet, while *Whrn*^wi/wi^ mice took several seconds to right themselves.

**Figure 2:**
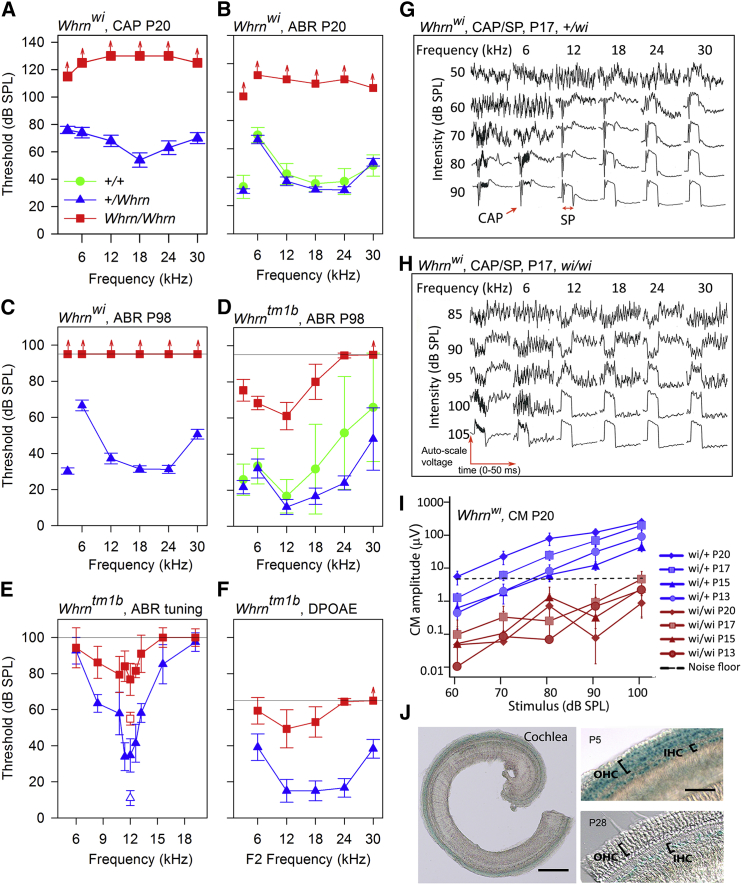
Auditory Electrophysiology and *Whrn* Expression in *Whrn*^wi/wi^ and *Whrn*^tm1b/tm1b^ Mice (A) Mean CAP threshold (±SD) in P20 *Whrn*^wi^ mice. (B) Mean ABR threshold (±SD) in P20 *Whrn*^wi^ mice. (C) Mean ABR threshold (±SD) in P98 *Whrn*^wi^ mice. (D) Mean ABR threshold (±SD) in P98 *Whrn*^tm1b^ mice. (E) Mean ABR masked tuning curves (±SD) in P98 *Whrn*^tm1b^ mice. Open symbols indicate mean (±SD) 12 kHz probe tone threshold. Filled symbols indicate mean (±SD) masked tuning thresholds. (F) Mean 2F1-F2 DPOAE threshold (±SD) in P98 *Whrn*^tm1b^ mice. Red arrows on symbols indicate maximum sound pressure level tested. (G) Waveforms from *Whrn*^+/wi^ mouse (P17) showing range of responses. CAP and SP responses are indicated. SP can be either negative (e.g., 12 kHz at 70 dB SPL) or positive (high frequencies, high intensities). (H) Waveforms from a *Whrn*^wi/wi^ mutant (P17), showing positive and negative SPs but no CAP. (I) Cochlear microphonic amplitudes plotted as a function of stimulus intensity for a 6 kHz tone. (J) LacZ staining in organ of Corti from *Whrn*^tm1b/tm1b^ mice at P5 (left: whole cochlea, right top: magnified) and P28 (bottom right). See also Figure S2.

We next evaluated cochlear physiology. In *Whrn*^wi/wi^ mice, recordings from the round window showed no compound action potentials (CAPs) even at the highest stimulus intensities used (Figure 2A). *Whrn*^wi/wi^ mutants also showed no auditory brainstem response (ABR) to the highest sound stimulus at P20 and P98 (Figures 2B and 2C). *Whrn*^+/wi^ mice had comparable ABR thresholds to *Whrn*^+/+^ mice, suggesting the mutation was completely recessive (Figure 2B). In contrast, 14-week-old mice homozygous for the *Whrn*^tm1b/tm1b^ allele showed a profound impairment only at higher frequencies (24–30 kHz) and only moderate impairment at lower frequencies (Figure 2D). Hearing loss in *Whrn*^tm1b/tm1b^ mice did not progress between 4 and 14 weeks (data not shown).

We assessed frequency tuning in *Whrn*^tm1b^ mice by measuring forward masked frequency tuning curves using a 12 kHz probe tone presented at 20 dB above threshold (Figure 2E). *Whrn*^+/tm1b^ mice produced a mean tuning curve with a sensitive tip located close to the 12 kHz probe tone. In contrast, *Whrn*^*t*m1b/tm1b^ mutants produced a mean tuning curve that was flat across frequencies (Figure 2E), suggesting impaired function of OHCs. OHC dysfunction in *Whrn*^tm1b/tm1b^ mutants was further indicated by measurements of 2F1-F2 distortion product otoacoustic emission (DPOAE) thresholds (Figure 2F). *Whrn*^+/tm1b^ mice had sensitive DPOAE thresholds for F2 frequencies, reflecting the shape of the ABR audiogram; *Whrn*^tm1b/tm1b^ mutants displayed moderate elevations in DPOAE threshold for F2 frequencies of 6–18 kHz, while DPOAEs from higher F2 frequencies were often not evoked even at the highest stimulus levels tested (Figure 2F).

HCs of *Whrn*^wi/wi^ mutant mice degenerate from around P21 (Holme et al., 2002), so we analyzed mice younger than this age to investigate HC function before the onset of degeneration. We recorded cochlear microphonics (an alternating voltage with the same frequency as the stimulus) that reflect OHC function and found that responses from *Whrn*^wi/wi^ mutants aged P13 to P20 were below detection limits, within the noise floor (Figure 2I). We also recorded summating potentials (Figures 2G and 2H), which are a sustained shift in potential for the duration of the 15-ms tone burst, and represent asymmetry of the receptor current between positive and negative phases of the acoustic stimulus (Dallos et al., 1972, Harvey and Steel, 1992). Summating potentials were detected in 27 of the 28 *Whrn*^wi/wi^ mutants studied, albeit at high stimulus intensities, suggesting that some HCs can depolarize in response to sound (example traces in Figure 2H). These data are consistent with previous reports where mechanotransduction currents were recorded from early postnatal HCs in *Whrn*^wi/wi^ mutants (Stepanyan et al., 2006).

Finally, we used lacZ staining to determine the endogenous expression pattern of WHRN-L in the inner ear. Since the lacZ reporter within the *Whrn*^tm1b^ allele is in the third intron (Figure S1A), it will be transcribed wherever WHRN-L is transcribed. The coding sequence of WHRN-S begins partway through exon 6, so the lacZ will not report WHRN-S expression. At P5, when stereocilia bundles are still developing, lacZ expression was detected in both IHCs and OHCs of the organ of Corti (Figure 2I). In the vestibular organs lacZ expression was very strong in a subpopulation of HCs: the extrastriolar region of the utricular macula (Figures S2A and S2C) and the peripheral zone of the crista ampularis (Figures S2B and S2D). Conversely, lacZ expression was not detected in the striolar and central zones (Figure S2, arrowheads). At P28, when HCs are mature, the pattern of lacZ expression in the vestibular organs remained consistent (Figure S2), but lacZ was no longer detected in OHCs of the organ of Corti (Figure 2I). These data suggest that endogenous expression of WHRN-L is high in cochlear OHCs in the early postnatal period but declines after functional maturity is reached.

### WHRN-S Expression Is Necessary and Sufficient for Normal IHC Stereocilia Length, but Both WHRN-S and WHRN-L Are Required for Normal OHC Stereocilia Bundle Morphology

**Figure 3:**
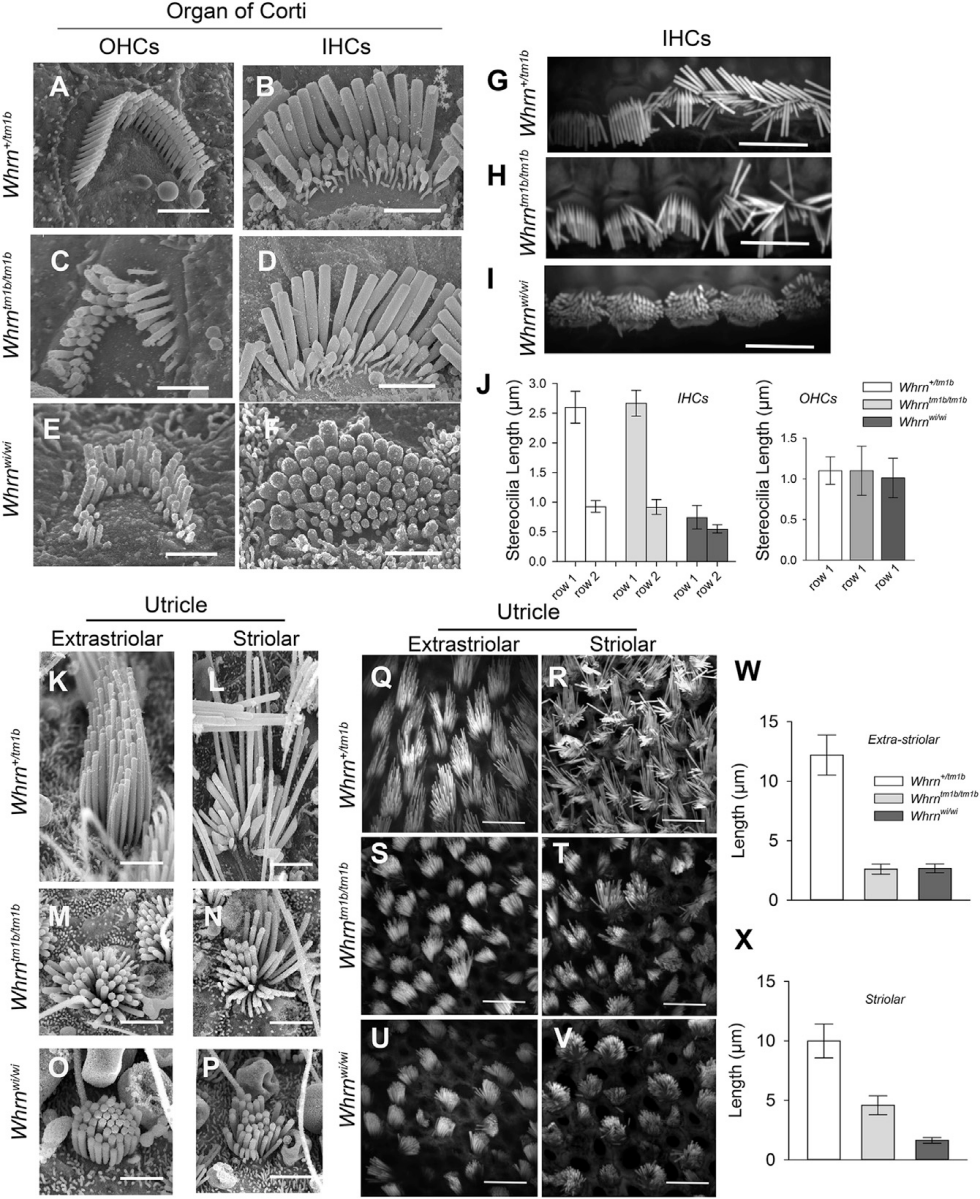
Differential *Whrn* Isoform Expression Affects Stereocilia Bundle Morphology (A and B) Scanning electron microscopy (SEM) images of OHCs (A) and IHCs (B) from P21 *Whrn*^+/tm1b^ mice. Scale bars, 2 μm. (C and D) SEM images of OHCs (C) and IHCs (D) from P21 *Whrn*^tm1b/tm1b^ mice. Scale bars, 2 μm. (E and F) SEM images of OHCs (E) and IHCs (F) from P21*Whrn*^wi/wi^ mice. Scale bars, 2 μm. (G–I) Confocal fluorescence images of IHC stereocilia (labeled with phalloidin) from P10 *Whrn*^+/tm1b^ (G), *Whrn*^tm1b/tm1b^ (H), and *Whrn*^wi/wi^ (I) mice. Scale bars, 5 μm. (J) Stereocilia length (±SD) from the tallest (row 1) and middle (row 2) row of IHC bundles and the tallest row (row 1) of OHCs of *Whrn*^+/tm1b^, *Whrn*^tm1b/tm1b^, and *Whrn*^wi/wi^ mice. (K–P) SEM images of vestibular stereocilia bundles in the extrastriolar and striolar region of the utricle of P21 *Whrn*^+/tm1b^ (K and L), *Whrn*^tm1b/tm1b^ (M and N), and *Whrn*^wi/wi^ (O and P) mice. Scale bars, 2 μm. (Q–V) Confocal fluorescence images of vestibular stereocilia bundles in the extrastriolar and striolar region of the utricle in P10 *Whrn*^+/tm1b^ (Q and R), *Whrn*^tm1b/tm1b^ (S and T), and *Whrn*^wi/wi^ (U and V) mice. Scale bars, 5 μm. (W and X) Length of the tallest stereocilia (±SD) from extrastriolar (W) and striolar (X) vestibular bundles. See also Figure S3.

To determine whether the observed differences in WHRN isoform expression between the *Whrn*^wi/wi^ and *Whrn*^tm1b/tm1b^ mice led to differences in stereocilia bundle morphology, we used scanning electron microscopy (SEM) and confocal fluorescence microscopy to image stereocilia bundles in the organ of Corti. We found, as previously reported (Holme et al., 2002, Mburu et al., 2003, Mogensen et al., 2007), that IHC stereocilia in the *Whrn*^wi/wi^ mutant were abnormally short (Figures 3F and 3I) and OHC stereocilia bundles were rounded (“U” rather than “W” shaped) and had irregular spacing between stereocilia rows (Figure 3E) compared to littermate controls (Figures 3A and 3B). *Whrn*^tm1b/tm1b^ OHC stereocilia bundles had a similar phenotype to the *Whrn*^*wi/*wi^ mutants (Figure 3C). Strikingly, however, the morphology of *Whrn*^tm1b/tm1b^ IHC stereocilia bundles (Figures 3D and 3H) appeared normal (Figures 3B and 3G). We measured the lengths of stereocilia from IHCs and OHCs from wild-type, *Whrn*^tm1b/tm1b^, and *Whrn*^wi/wi^ mutants and found no significant difference between wild-type and *Whrn*^tm1b/tm1b^ mice (Figure 3J). This suggests that the expression of WHRN-S is necessary to maintain normal IHC stereocilia length at P21. Looking for intersterocilia links, we found lateral links between adjacent stereocilia in some *Whrn*^tm1b/tm1b^ OHC bundles (Figure S3). This was also consistent with lateral links that have been previously reported in OHC stereocilia of *Whrn*^wi/wi^ mice (Mogensen et al., 2007).

We next assessed uptake of FM1-43, a compound that permeates the stereocilia mechanotransducer channels (Gale et al., 2001), in cochlear HCs from *Whrn*^tm1b/tm1b^ and *Whrn*^wi/wi^ mice at P10 to determine whether their abnormal morphology affected mechanoelectrical transduction (MET). Uptake of FM1-43 was observed in both IHCs and OHCs of both *Whrn*^tm1b/tm1b^ and *Whrn*^wi/wi^ mice (Figures S3H and S3I). OHCs load more dye than IHCs, as previously reported (Gale et al., 2001). These data are consistent with our finding of summating potential responses in the *Whrn*^wi^ mutants and previous reports of MET recordings from *Whrn*^wi^ mutant OHCs (Stepanyan et al., 2006), suggesting that some transduction can occur in *Whrn* mutant HCs at high stimulus levels.

### Vestibular Stereocilia Bundles Show Distinct Morphologies in *Whrn*^wi/wi^ and *Whrn*^tm1b/tm1b^ Mice

We analyzed stereocilia in the vestibular organs in each mutant line to determine whether bundle morphology was responsible for the observed differences in vestibular phenotype between them. In *Whrn*^wi/wi^ mutants stereocilia were consistently markedly shorter (Figures 3O, 3P, 3U, 3V, S3C, and S3F) than in controls (Figures 3K, 3L, 3Q, 3R, S3A, and S3D), across all HCs. In the *Whrn*^tm1b/tm1b^ mice, however, we observed interesting regional differences in bundle morphology: while stereocilia bundles in the extrastriolar region of the utricular and saccular maculae were short (Figures 3M and 3S) as in the *Whrn*^wi/wi^ mutants (Figures 3O and 3U), bundles in the striolar region were more variable, with longer stereocilia and a closer to normal staircase organization (Figures 3N and 3T). We quantified this by measuring the length of tallest stereocilia in extrastriolar (Figure 3W) and striolar (Figure 3X) bundles in *Whrn*^+/tm1b^, *Whrn*^tm1b/tm1b^, and *Whrn*^wi/wi^ mice. In both *Whrn*^tm1b/tm1b^ and *Whrn*^wi/wi^ mutants, the tallest stereocilia in extrastriolar bundles showed an ∼80% reduction in length compared to *Whrn*^+/tm1b^ mice (Figure 3W). In the striolar region, the tallest stereocilia in *Whrn*^wi/wi^ mutants again showed an ∼80% reduction in length compared to *Whrn*^+/tm1b^ mice, but in *Whrn*^tm1b/tm1b^ mutants the length reduction was closer to ∼50% (Figure 3X). The regional differences in stereocilia morphology were particularly pronounced in the crista ampularis of the *Whrn*^tm1b/tm1b^ mutants (Figures S3A–S3F), where bundles in the central regions were much shorter (Figure S3B) than heterozygous controls (Figure S3A), but bundles in the peripheral zone (Figure S3E) had morphology, comparable to control bundles (Figure S3D). These data are consistent with a similar phenotype reported in *Whrn*^neo/neo^ mice (Mathur et al., 2015a). Because the stereocilia in the crista ampularis can be up to 100 μm long, it was not possible to measure the precise lengths of stereocilia in this organ. However, the presence of regions in all vestibular organs of HCs with stereocilia morphology that was either normal or closer-to-normal (compared with *Whrn*^wi/wi^ mutants), likely explains the lack of overt vestibular phenotype in the *Whrn*^tm1b/tm1b^ mutants.

### WHRN-S and WHRN-L Have Distinct Localizations within Stereocilia in Auditory Hair Cells

**Figure 4:**
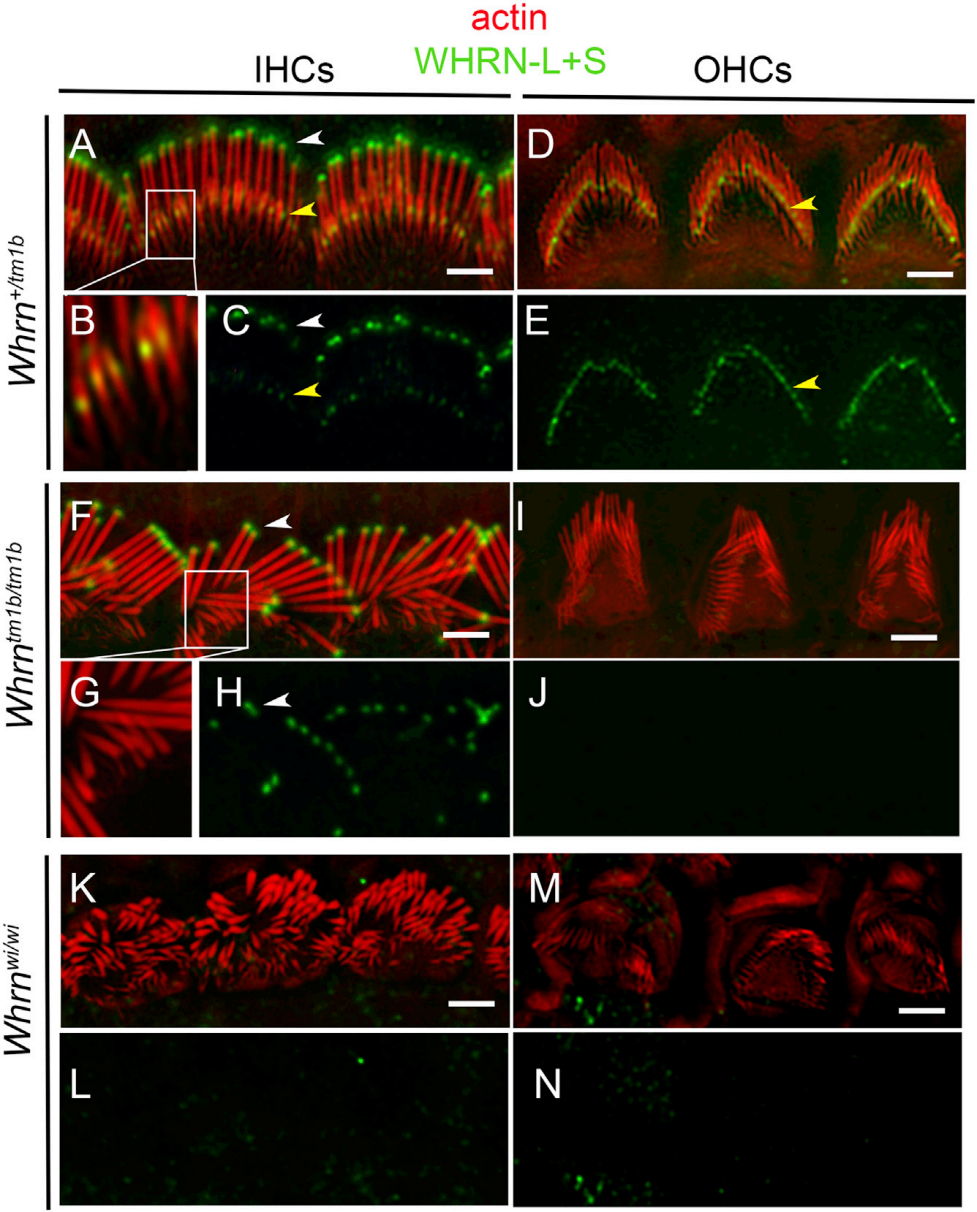
*Whrn* Isoforms Show Distinct Localizations within Stereocilia (A–C) WHRN (green) localizes in IHC stereocilia (red) to two distinct regions in P10 *Whrn*^+/tm1b^ mice: stereocilia tips of the tallest row (white arrowhead) and proximal to the tips of the shorter row of stereocilia (yellow arrowhead). Boxed region in (A) is shown in high-magnification (B). (D and E) WHRN (green) localizes in OHC stereocilia (red) predominantly to the shorter row of stereocilia in P10 *Whrn*^+/tm1b^ mice. (F–H) WHRN (green) localizes in IHC stereocilia (red) to stereocilia tips of the tallest row (F, arrowhead) in *Whrn*^tm1b/tm1b^ mice. WHRN localization to shorter stereocilia is not detected. Boxed region magnified in (G). (I and J) WHRN (green) localization is not detected in OHC stereocilia (red) in *Whrn*^tm1b/tm1b^ mice (I). (K–N) WHRN label in *Whrn*^wi/wi^ mutant stereocilia. WHRN (green) is not detected in *Whrn*^wi/wi^ stereocilia (red) from IHCs (K and L) or OHCs (M and N). Scale bars, 2 μm. See also Figure S4.

To explore the molecular mechanisms underlying the differences in stereocilia length between *Whrn*^tm1b/tm1b^ and *Whrn*^wi/wi^ mutants, we investigated the precise localization of WHRN-S and WHRN-L within stereocilia. We used a custom antibody (PB584) against an epitope just upstream of the PDZ3 domain (Figure 1A), which labels both WHRN-L and WHRN-S (Figure S1). Therefore, both isoforms should be labeled in stereocilia of control mice, only WHRN-S in *Whrn*^tm1b/tm1b^ mice, and no immunoreactivity is expected in the *Whrn*^wi/wi^ mutants where both isoforms are ablated. We labeled all three mouse lines at P10 and found that in control mice, consistent with previous reports, WHRN localized at stereocilia tips of the tallest row in IHCs (Figures 4A and 4C, white arrowhead). We also detected a distinct distribution of WHRN, proximal to the tips of shorter stereocilia (Figures 4A–4C, yellow arrowhead). In OHCs at P10, the localization of WHRN at the stereocilia tips of the tallest row was not detected (some localization at the tips of OHCs was detected at P8; see Figures S4D and S4E), while its localization proximal to the tips of shorter stereocilia persisted (Figures 4D and 4E). To pinpoint this latter localization in IHC (Figures S4A–S4C) and OHC stereocilia of wild-type mice (Figures S4D–S4F), we used high-resolution spinning disc confocal microscopy (Figures S4A and S4D) as well as SIM (Figures S4B–S4F). We used the SIM images to track this localization of WHRN relative the tip and base of the second row of stereocilia (Figure S4G) and found localization within the upper half of the shorter stereocilia. Interestingly, in *Whrn*^tm1b/tm1b^ mice, which express WHRN-S but not WHRN-L, PB584 labeled the tips of the tallest row of stereocilia in IHCs (Figures 4F and 4H, white arrowhead), but localization in shorter stereocilia was no longer detectable (Figures 4F–4H). The absence of WHRN labeling in the shorter row, which was also noted in OHCs (Figures 4I and 4J), suggests that this particular localization can be attributed to WHRN-L. The localization of WHRN-L proximal to the tips of the second row of stereocilia was further verified (Figure S4H) using an isoform-specific antibody PB595, (validated in Figure S1), directed to the N terminus of WHRN-L (Figure 1A). Antigen retrieval was needed for stereocilia labeling with PB595, suggesting that the epitope (between PDZ1 and PDZ2; Figure 1A) may be sterically challenging to access. Despite higher background fluorescence as a result of antigen retrieval, no stereocilia tip localization was detected with PB595 (Figure S4H, white arrows). No PB595 immunoreactivity was detected in tm1b/tm1b mice (Figures S4I and S4J). No WHRN immunoreactivity was detected in the IHCs or OHCs of *Whrn*^wi/wi^ mutants, in which both WHRN-S and WHRN-L are ablated (Figures 4K–4N). Together, these data indicate that WHRN-S and WHRN-L have distinct localizations within stereocilia.

### WHRN-S Colocalizes with EPS8 and Both Are Required for Normal Stereocilia Length

**Figure 5:**
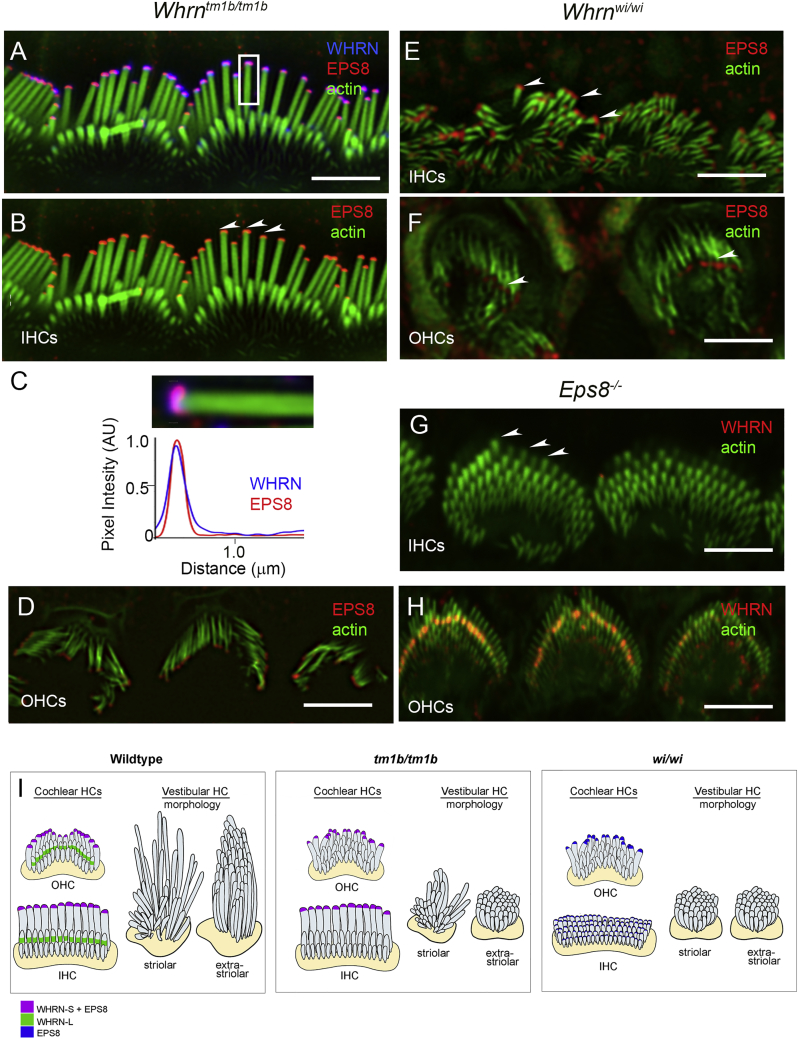
WHRN-S Localization at Stereocilia Tips Overlaps with EPS8 and Both Proteins Are Required for Normal Stereocilia Elongation (A and B) Localization of WHRN and EPS8 to stereocilia tips. SIM image of IHC stereocilia (green) from P11 *Whrn*^tm1b/tm1b^ mice. WHRN-S (blue) and EPS8 (red) colocalize at stereocilia tips (arrowheads). (C) High mag of stereocilium from box in (A) and corresponding fluorescence intensity profile (below). (D) EPS8 (red) in OHC stereocilia (green) from P11 *Whrn*^tm1b/tm1b^ mice. (E and F) EPS8 (red) immunofluorescence in stereocilia (green) from P10 *Whrn*^wi/wi^ mutants shows stereocilia tip localization (arrowheads) in IHCs (E) and OHCs (F). (G and H) WHRN (red) immunofluorescence in stereocilia (green) from P6 *Eps8* null mouse. No stereocilia tip localization observed in IHCs (G) or OHCs (H). WHRN-L labeling is still detected in OHCs (H). (I) Schematic summarizing WHRN isoform and EPS8 localization in stereocilia of cochlear hair cells, and vestibular stereocilia bundle morphology in wild-type, *Whrn*^tm1b/tm1b^, and *Whrn*^wi/wi^ mice. Scale bars, 2 μm.

EPS8 is an actin regulatory protein that has been shown to be transported, along with WHRN, by MYO15a to stereocilia tips, where it has been implicated in stereocilia elongation (Manor et al., 2011). To investigate the specific roles of WHRN-S and WHRN-L, we asked whether the distribution of EPS8 varied in stereocilia from *Whrn*^wi/wi^ and *Whrn*^tm1b/tm1b^ mice. In *Whrn*^tm1b/tm1b^ mice, EPS8 localized to the tallest row of stereocilia in IHCs (Figures 5A and 5B) and OHCs (Figure 5D) and colocalized strongly with WHRN-S (Figures 5A–5C), while no WHRN-L was detected. In *Whrn*^wi/wi^ mice, EPS8 localization to stereocilia tips at least partially persisted in both IHCs (Figure 5E, arrowheads) and OHCs (Figure 5F, arrowheads), consistent with previous reports (Manor et al., 2011). Strikingly, however, in *Eps8* null mutants (Offenhäuser et al., 2006) that also have short stereocilia (Manor et al., 2011), we found that WHRN-S immunofluorescence was not detected at the tips of the tallest row of stereocilia in IHCs (Figure 5G) or OHCs (Figure 5H).WHRN-L localization proximal to the tips of the shorter rows was maintained in OHCs (Figure 5H), but not detected in IHCs (Figure 5G). Together, these data show that in the absence of EPS8, WHRN-S is not able to properly localize to stereocilia tips. Furthermore, that *Whrn*^wi/wi^ mice have short stereocilia despite the presence of EPS8 at stereocilia tips suggests that in HCs, both EPS8 and WHRN-S are required to modulate stereocilia elongation.

## Discussion

In the current study, we show that the major WHRN isoforms, WHRN-S and WHRN-L, show differential expression within stereocilia and across HC type. While lack of both isoforms results in abnormally short stereocilia across HCs, we find that WHRN-S alone is sufficient to maintain normal stereocilia bundle morphology in IHCs and a subset of vestibular HCs. Accordingly, mice expressing WHRN-S have a milder auditory phenotype than mice lacking both WHRN-S and WHRN-L (Holme et al., 2002) and also show no overt vestibular problems. These findings are consistent with mapped pathogenic variants in human patients and their respective phenotypes: DFNB31 patients, who suffer from profound prelingual sensorineural hearing loss (Mustapha et al., 2002), have mutations within exons 10 and 11 of *DFNB31* (Mburu et al., 2003, Mustapha et al., 2002, Tlili et al., 2005), which affect the PDZ3 domain of both WHRN-L and WHRN-S, likely resulting in truncated, non-functional proteins. Mutations in patients with USH2D, who have milder and more variable hearing abnormalities, have been localized to exons 1 and 2 and intron 2 of *DFNB31* (Audo et al., 2011, Besnard et al., 2012, Ebermann et al., 2007), which affect PDZ1 and PDZ2 of WHRN-L, but likely have no impact on the expression of WHRN-S. Our findings are also consistent with the report of a different mutation targeting exon 1 of *Whrn* in the mouse, where IHCs appeared normal and OHCs showed similar abnormalities as we report here (Yang et al., 2010). While genetic analysis does not preclude a role in HC function for WHRN-M, which contains one and part of another PDZ domain, the stereocilia phenotype in the *Whrn*^wi/wi^ mouse manifests across all HCs despite the predicted expression of WHRN-M in this line.

That *Whrn*^tm1b/tm1b^ mutants had normal IHC morphology, tip links, and a lack of profound deafness, suggests their IHCs are at least partly functional. Conversely, abnormal OHC morphology, poor frequency tuning, and raised DPOAE thresholds (40 dB higher than littermate controls) suggest impaired OHC function. Indeed, the electrophysiological responses of *Whrn*^tm1b/tm1b^ mutants are very similar to those reported for the prestin mutant (Cheatham et al., 2004), which are believed to have abnormal OHC but normal IHC function.

WHRN has previously been described as localizing to the stereocilia tip and base region (Mathur et al., 2015b, Michalski et al., 2007, Zou et al., 2014). Using immunofluorescence at sub-diffraction limit resolution afforded by SIM, we found that while WHRN-S localizes to stereocilia tips (consistent with previous reports), WHRN-L localizes toward the middle of the stereocilia rather than the base. This localization leads us to propose that WHRN-L may have a role in scaffolding inter-stereociliary links such as lateral links or horizontal top-connectors, that join adjacent stereocilia within and between rows (Hackney and Furness, 2013). In such a scenario, the lack of WHRN-L may result in weakened scaffolding of inter-stereociliary links and compromised OHC function. We detected some lateral links between adjacent stereocilia in P28 *Whrn*^tm1b/tm1b^ mutants (Figure S3), although this does not preclude a potential role for WHRN-L as a scaffold.

In the *Eps8* null mutant, WHRN-S does not localize properly to stereocilia tips but WHRN-L localization is unchanged, suggesting that WHRN-S requires EPS8 for normal stereocilia tip localization. WHRN-L localization is diminished in *Eps8* null IHCs, suggesting that WHRN-L and EPS8 interactions may also be hair cell-specific. Similarly, it is interesting that the absence of WHRN-L results in shorter stereocilia only in certain vestibular hair cells. Together these observations, summarized in Figure 5I, suggest that there are multiple pathways or networks, comprising distinct molecular players, for stereocilia length regulation in different cell types. In line with isoform-specific functions described in this study, two isoforms of MYO15 were recently shown to selectively traffic to different stereocilia rows, where they have independent functions (Fang et al., 2015). Possible mechanisms underlying the differential targeting of WHRN isoforms include: (1) specific combinations of MYO15/WHRN isoforms form the basis for targeted trafficking of the different complexes within stereocilia; (2) WHRN-L, with its additional PDZ domains, is sterically restricted from being trafficked to stereocilia tips; and (3) WHRN-S requires active transport by a molecular motor to target stereocilia tips, while passive transport may be sufficient for the more proximal localization of WHRN-L. While mechanistic details remain to be elucidated, the data thus far paint a picture of the WHRN/EPS8/MYO15 complex as having multiple roles regulated by its constituent isoforms. It is becoming increasingly clear, from these studies and others, that many stereocilia-associated genes have multiple protein-coding products with not only distinct, but also very different functions. Additionally, to obtain a holistic picture of the molecular basis underlying stereocilia bundle formation, maintenance, and function, expression profiles of even well-known stereocilia-associated proteins must be considered across HC-type and their localizations carefully assessed within the stereocilia bundle.

## Experimental Procedures

### Production and Genotyping of *Whrn* Mutants

The *Whrn*^tm1b^ allele was generated by mating *Whrn*^tm1a(KOMP)Wtsi^ mice, produced at the Wellcome Trust Sanger Institute, to *Hprt*^Tg(CMV-Cre)Brd^ mice that express Cre recombinase widely. The *Whrn*^wi/wi^ allele originated on an undefined genetic background and has since been maintained within a closed colony for over 30 years. Details are in the Supplemental Experimental Procedures.

### RNA Extraction and RT-PCR

RNA was extracted from cochlear and vestibular organs from mouse inner ears using QIAshredder columns (QIAGEN, cat. no. 79654) and the RNeasy mini kit (QIAGEN, cat. no. 74104), or the Lexogen SPLIT kit (Lexogen, cat. no. 008.48). RNA concentration was measured using a Nanodrop spectrophotometer (ND-8000). cDNA was made using Superscript II Reverse Transcriptase (Invitrogen, cat. no. 11904-018) before Sanger sequencing. Details in the Supplemental Experimental Procedures.

### Immunofluorescence Microscopy

Organ of Corti tissue was labeled using antibodies against mouse WHRN (PB384 and PB595) and mouse EPS8 and viewed in a Nikon inverted fluorescence microscope, outfitted with a spinning disk confocal scan head or an N-SIM Super Resolution System, 100× Apo TIRF 1.49 NA objective, and a CMOS camera. NIS-Elements imaging software was utilized for image acquisition and reconstruction. Details are in the Supplemental Experimental Procedures.

### Statistical Methods

Means and SDs were calculated using Microsoft Excel.

## Author Contributions

S.E. designed experiments and collected and analyzed scanning electron microscopy and immunolocalization data; M.A.L. carried out the molecular analysis; N.I., M.A.L., and J.C.P. carried out the ABR analysis; M.J.C.R. carried out the round window recordings; J.C.P. produced the tm1b allele; and R.C. and B.K. labeled and imaged the *Eps8* mutant sample and validated antibodies in COS7 cells. All authors analyzed the results. K.P.S. led the project and S.E. wrote the paper and generated the figures. All authors contributed to the final version.

## Accession Numbers

The accession numbers for the isoforms reported in this paper are GenBank: AY739121.1 and GenBank: NM_001008795.1.

## Supplemental Information

Document S1. Supplemental Experimental Procedures, Figures S1–S4, and Tables S1 and S2

Document S2. Article plus Supplemental Information
