# Supplementary material for: Alternative Splice Forms Influence Functions of Whirlin in Mechanosensory Hair Cell Stereocilia
Source: Cell Rep. 2016 Apr 21;15(5):935–43. doi: 10.1016/j.celrep.2016.03.081 (PMC4859837; doi:10.1016/j.celrep.2016.03.081)
Supplement: Document S1. Supplemental Experimental Procedures, Figures S1–S4, and Tables S1 and S2 [file mmc1.pdf]

Cell Reports, Volume *15*

Supplemental Information

Alternative Splice Forms Influence Functions of Whirlin in Mechanosensory Hair Cell Stereocilia

Seham Ebrahim, Neil J. Ingham, Morag A. Lewis, Michael J.C. Rogers, Runjia Cui, Bechara Kachar, Johanna C. Pass, and Karen P. Steel

Figure S1

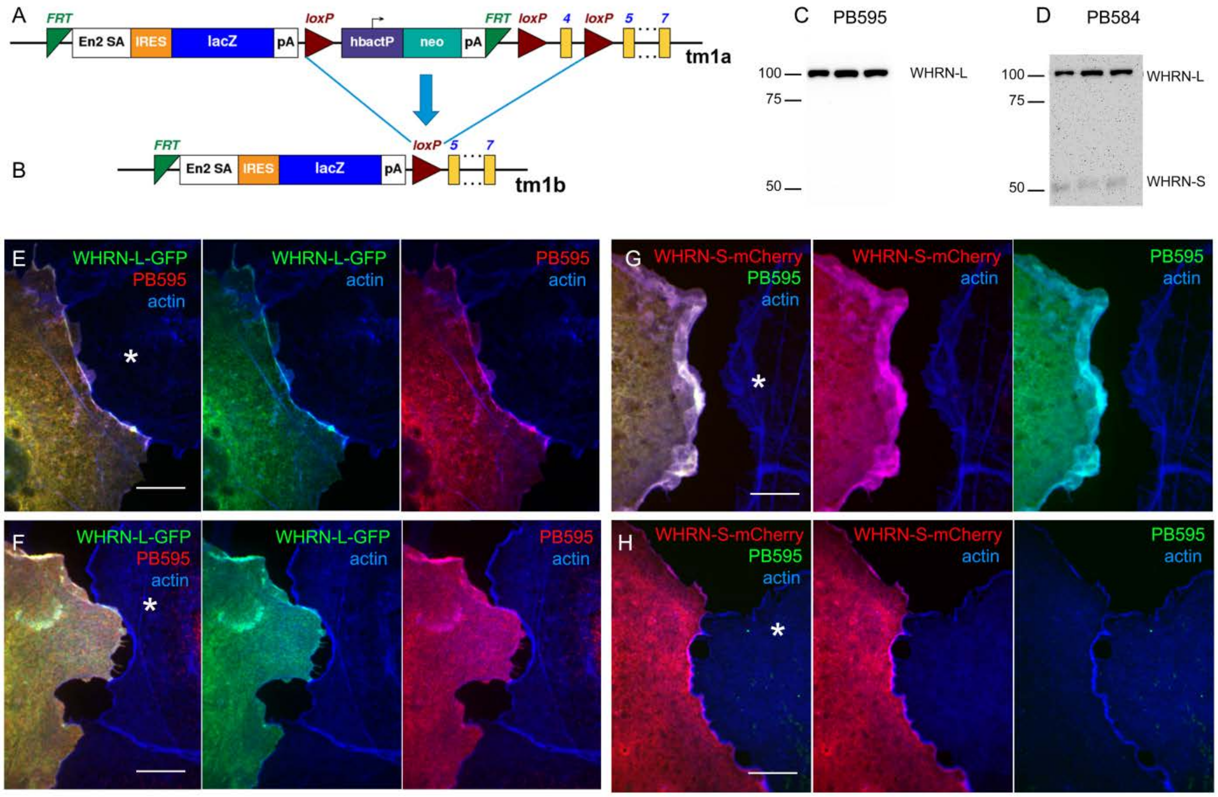

Figure S1. - Generation of *Whrn*^{tm1a} and *Whrn*^{tm1b} alleles, and validation of anti-WHRN antibodies. (A and B) Generation of *Whrn*^{tm1a} and *Whrn*^{tm1b} alleles. (A) Diagram representing the *Whrn*$^{tm1a(KOMP)Wtsi}$ allele. Exons are indicated by yellow rectangles. The large selection cassette is surrounded by FRT sites (green triangles) and LoxP sites (red triangles) flanking exon 4 allow excision by exposure to Cre recombinase. Transcription of the tm1a allele is inhibited by the large extraneous DNA insertion, acting in a similar way to a gene-trap allele. (B) The tm1b allele is derived from the tm1a allele by crossing with *Hprt*$^{Tg(CMV-Cre)Brd}$ mice, which express Cre recombinase, leading to recombination between two LoxP sites, thereby deleting exon 4 and part of the inserted cassette. (C and D) Western blots from retina and inner ear tissue, combined, from three *Whrn*$^{+/tm1b}$ mice probed with PB595 (C) and PB584 after stripping (D). (E - H) Validation of anti-WHRN antibodies. COS7 cells were transfected with WHRN-L-GFP (green) and labeled with PB584 (E) or PB595 (F), an anti-rabbit secondary antibody conjugated to Alexa Fluor 568 (red), and phalloidin to mark actin (blue). Colocalization (yellow signal) between WHRN-L-GFP and PB584 (E) or PB595 (F) in the transfected cell confirms antibody specificity. Lack of red signal in the untransfected cells (asterisks) confirms antibody selectivity. COS7 cells were also transfected with WHRN-S-mCherry (red) and labeled with PB584 (G) or PB595 (H), an anti-rabbit secondary antibody conjugated to Alexa Fluor 488 (green), and phalloidin (blue). Colocalization (yellow signal) between WHRN-S-mCherry and PB584 (G) in the transfected cell confirms antibody specificity for WHRN-S. No green signal was observed in transfected cells labeled with PB595 (H), confirming that PB595 does not label WHRN-S. Scale bar = 10 µm. **Related to Figure 1 and 4**

Figure S2

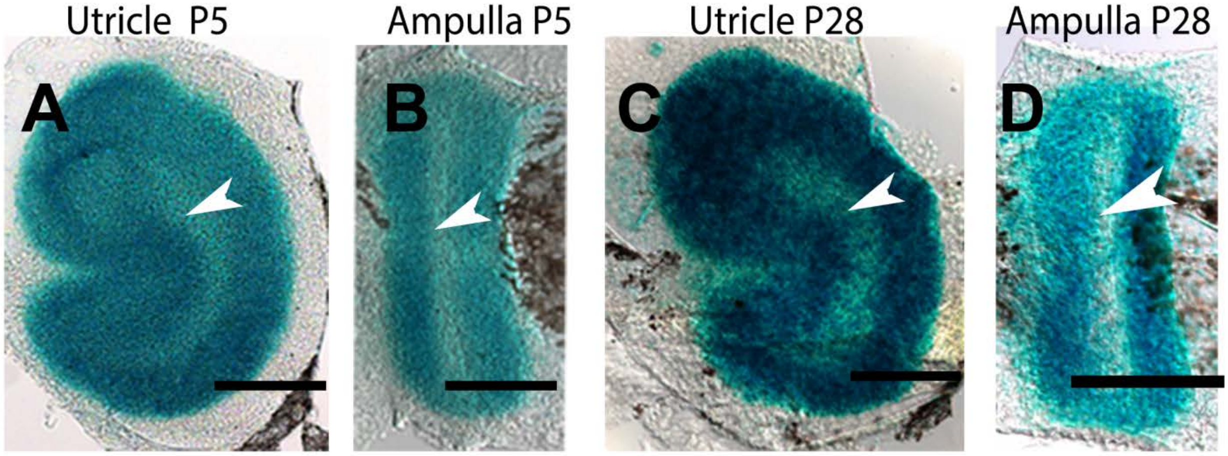

Figure S2. LacZ staining in vestibular sensory epithelia from *Whrn*$^{tm1b/tm1b}$ mice. At both P5 (A and B) and P28 (C and D) the label is weaker in the striolar region of the utricle and the central zone of the ampullae (arrowheads). Scale bars = 500 µm. **Related to Figure 2.**

Figure S3

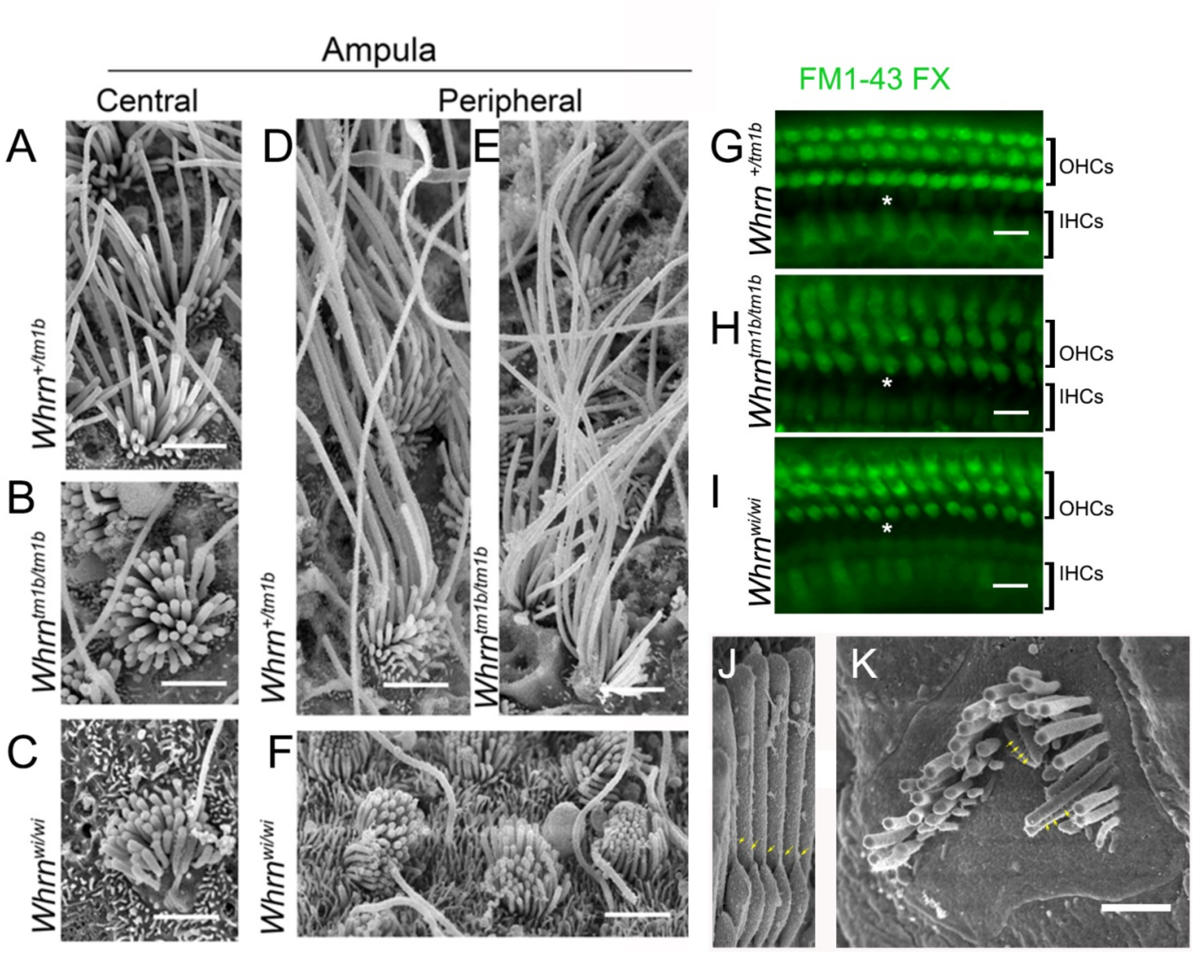

Figure S3. Cristae ampularis bundle morphology, and FM1-43 uptake in *Whrn*$^{tm1b/tm1b}$ and *Whrn*$^{wi/wi}$ mutants. Examples of stereocilia bundle morphology in the central zone in *Whrn*$^{+/tm1b}$ (A), *Whrn*$^{tm1b/tm1b}$ (B) and *Whrn*$^{wi/wi}$ (C) mice at P21. Examples of peripheral zone bundles in *Whrn*$^{+/tm1b}$ (D), *Whrn*$^{tm1b/tm1b}$ (E) and *Whrn*$^{wi/wi}$ (F) mice. (G-I) FM1-43 dye (green) applied to the organ of Corti showed loading in both IHCs and OHCs of *Whrn*$^{+/tm1b}$ mice (G) as well as *Whrn*$^{tm1b/tm1b}$ (H) and *Whrn*$^{wi/wi}$ (I) mutants. Support cells do not show any dye uptake (white asterisks). (J and K) Some inter-stereocilia links are present in the *Whrn*$^{tm1b/tm1b}$ mutant. SEM of IHC stereocilia from *Whrn*$^{tm1b/tm1b}$ mutants (P28) showing the presence of tip links (J, yellow arrows). SEM of OHC stereocilia from *Whrn*$^{tm1b/tm1b}$ mutants (P28) showing the presence of lateral links between adjacent stereocilia (K, yellow arrows). Scale bars A-F= 2 µm; G-1 = 10 µm; K = 1 µm. **Related to Figure 3.**

Figure S4

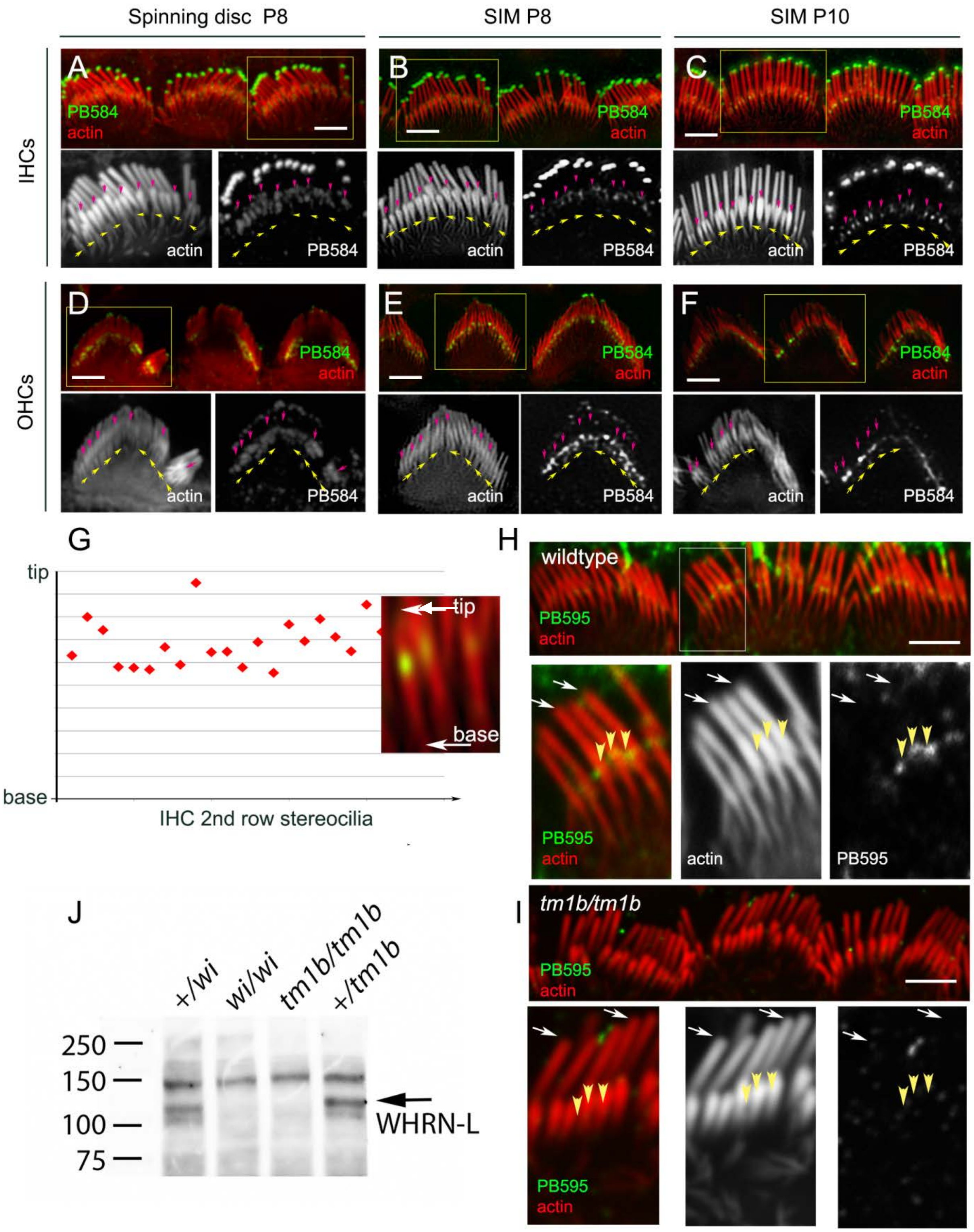

Spinning disc P8
SIM P8
SIM P10
IHCs
OHCs
A
B
C
D
E
F
PB584
actin
G
tip
base
IHC 2nd row stereocilia
H
wildtype
PB595
I
tm1b/tm1b
J
+/wi
wi/wi
tm1b/tm1b
+/tm1b
250
150
100
75
WHRN-L

Figure S4. WHRN-L localization in IHC and OHC stereocilia. (A-C) IHC stereocilia (red) labeled with PB584 (green) to detect both WHRN-L and WHRN-S, and imaged using conventional spinning disc microscopy at P8 (A) and super-resolution SIM at P8 (B) and P10 (C). Pink arrowheads point to tips of second row stereocilia, and yellow arrowheads point to stereocilia bases. (D-F) OHC stereocilia (red) labeled with PB584 (green) to detect both WHRN-L and WHRN-S, and imaged using conventional spinning disc microscopy at P8 (D) and SIM at P8 (E) and P10 (F). Pink arrowheads point to tips of second row stereocilia, and yellow arrowheads point to stereocilia bases. (G) Plot showing the localization of WHRN-L (red dots) relative to the tip and base of second row stereocilia. Inset-magnified view of second row stereocilia with WHRN-L labeled (green) and tip and base highlighted (white arrows). (H and I) IHC stereocilia (red) from wildtype (H) and tm1b/tm1b (I) mice at P10 labeled with PB595, an antibody specific to WHRN-L (green). White arrows point to tips of tallest row of stereocilia. Arrowheads points to region of WHRN-L localization, proximal to the tips of the second row of stereocilia. (J) Western blot of brain tissue from +/wi, wi/wi, +/tm1b and tm1b/tm1b mice probed with PB595 (against WHRN-L). Scale bars= 2 µm. **Related to Figure 4.**

Table S1- WHRN isoforms with corresponding Ensembl and GenBank Accession Numbers

Isoform	Ensembl Transcript ID	Genbank IDs
WHRN-L	ENSMUST00000084510	NM_001008791
WHRN-L	ENSMUST00000107393	NM_001008792
WHRN-L	ENSMUST00000063650	NM_028640
WHRN-L	ENSMUST00000102867	NM_001008793
WHRN-M	ENSMUST00000063672	AK157955.1
WHRN-S	ENSMUST00000095038	NM_001008794
WHRN-S	ENSMUST00000119294	NM_001008795.1
WHRN-S	ENSMUST00000095037	NM_001008796
WHRN-N	ENSMUST00000133425	NM_001276371
WHRN-N	ENSMUST00000144965	BM940881.1
WHRN-S	ENSMUST00000155058	AY739121.1

Table S2- Primers used to amplify *Whrn* protein-coding transcripts

Exon	Primer name	Sequence
ENSMUSE00000603856, ENSMUSE00001218627	1F	CTGCAGCCCCAGGATTTTAC
ENSMUSE00000662852	2F	ACGATAAATCTCTAGCCCGGG
ENSMUSE00001218627	3F	AGTACCCTCCACCTCCTGC
ENSMUSE00001236620	4F	CACACAGAAGAAGGGACAAGC
ENSMUSE00000789315	f	GTAGGAGATGCGAGCACTTTGTACGC
ENSMUSE00001278579	5F	CGCCACCATGATGTACTACC
ENSMUSE00000747140	1R	GTGTGAGACCTTCTTGCTGG
ENSMUSE00000776975	2R	GACAGACTCTCCCATGGCC
ENSMUSE00001268183	3R	GTCTTGGGGTGAGATGATGC
ENSMUSE00000222478	5R	TCTTAGATGCCCACTGTCCC
ENSMUSE00001245215	d	GTTAACCAGGGCCAGGTGGGTGTC
ENSMUSE00001245215	6R	GTCATGACCTTGAGCCAGG
ENSMUSE00001271849	7R	CTGCACTTTTCCTCACACGG
ENSMUSE00001295720	8R	TGCCTTCCTTCCCTTCACC

Isoform	Specific primer sets
WHRN-N	2F/1R, 3F/2R
WHRN-M	5F/5R
WHRN-S	f/d, 1F/3R
WHRN-L	3F/6R, 3F/7R, 4F/6R, 4F/7R, 4F/8R

Supplemental Experimental Procedures

Ethics statement

Mouse studies were carried out in accordance with UK Home Office regulations and the UK Animals (Scientific Procedures) Act of 1986 under Home Office licences, and the study was approved by both the Wellcome Trust Sanger Institute and the King's College London Ethical Review Committees. Mice were culled using methods approved under these licences to minimize any possibility of suffering. *Eps8* null mice experiments were conducted in accordance with animal protocols approved by the NIH Animal Care and Use Committee (Protocol #1264).

Production and genotyping of *Whrn* mutants

Whrn$^{tm1a(KOMP)Wtsi}$ mice were produced at the Wellcome Trust Sanger Institute and carry a knockout-first conditional-ready allele [1, 2]. The mice were maintained in individually-ventilated cages at a standard temperature and humidity and in specific pathogen-free conditions on a C57BL/6N genetic background. For genotyping, DNA was extracted from ear-clips and used for short range PCR. For the tm1a allele, wild type primers were: forward GCTCATTTGCCCATTGTTTTC, reverse CTGAAGTCCCCCACCAGAAG, product size of 505bp. The mutant allele shares the forward primer with wild type and the reverse primer TCGTGGTATCGTTATGCGCC to produce a 196bp product. The wild type reaction spans exons 3-4 and fails in the presence of the cassette as the sequence is too large to be amplified by short range PCR. *Whrn*$^{tm1a/tm1}$a mutants showed ABR response thresholds very similar to those of *Whrn*$^{tm1b/tm1b}$ (data not shown), so the latter allele was used for the remaining experiments.

The *Whrn*^{tm1b} allele was generated by mating mice carrying the tm1a allele to *Hprt*$^{Tg(CMV\text{-}Cre)Brd}$ mice which express Cre recombinase widely, resulting in recombination between LoxP sites in the *Whrn*^{tm1a} allele. The tm1b allele was genotyped using the same primers used for the tm1a allele. The CMV-Cre allele was bred out of the colony before phenotypic characterization. The primers used to detect the wild type sequence at the *Hprt* locus were: forward CTTTCCTCATGCCCCAAAATCTTAC and reverse ATGTAATCCAGCAGGTCAGCAAGA to produce a 311 bp product. For the mutant *Hprt*$^{Tg(CMV\text{-}Cre)Brd}$ allele , the forward primer is shared with the wild type and the reverse primer was GCTATCAGGACATAGCGTTGGCTAC, giving a 700 bp product. Primers for LoxP (forward ATCCGGGGGTACCGCGTCGAG and reverse ACTGATGGCGAGCTCAGACC) and tm1b (forward CGGTCGCTACCATTACCAGT, reverse is shared with LoxP) were used to confirm recombination and conversion into a tm1b allele. After complete conversion to a tm1b allele the LoxP reaction fails as only one LoxP site remains. In the tm1a allele the LoxP PCR product is between 800bp and 1kb and is reduced to 130bp if only the critical exon is removed (see Figure 1A, B). When recombination removes only the *neo* gene and not exon 4, the tm1b reaction produces a 1kb product. Following full conversion to a tm1b allele, where both the *neo* and exon 4 are removed, the tm1b reaction product size is 380bp.

The *Whrn*$^{wi/wi}$ allele originated on an undefined genetic background and has since been maintained within a closed colony for over 30 years. Round window recordings were carried out using mice maintained in a conventional facility at a standard temperature and humidity using homozygous mutants and heterozygous littermate controls. All other experiments were carried out after rederivation into specific pathogen-free conditions in individually-ventilated cages. Primers for genotyping were: wild type forward ATGAAAGTGCCACGGAGATG and wild type reverse TGCACTCTGTCCCTGACTCC, and the mutant reverse CCAGAGCTGGACAACCATAC which shares the wild type forward primer, giving a wild type product size of 476bp and a mutant allele product size of 678bp. The wild type reverse primer is within the deleted region, so a product should not be generated for the mutant allele. The mutant reverse is on the other side of the deletion and should not generate a product for extension times below 45 s.

RNA extraction and RT-PCR

Cochlear and vestibular organs from mouse inner ears were dissected in RNAlater (Ambion, cat. no. AM7024) or phosphate buffered saline (PBS), pH 7.4 and stored in RNAlater. RNA was extracted using QIAshredder columns (QIAgen, cat. no. 79654) and the RNeasy mini kit (QIAgen, cat. no. 74104), or the Lexogen SPLIT kit (Lexogen,

cat. no, 008.48), following the manufacturer's instructions. RNA concentration was measured using a Nanodrop spectrophotometer (ND-8000). cDNA was made using Superscript II Reverse Transcriptase (Invitrogen, cat. no. 11904-018) after treatment with DNAse 1 (Sigma, cat.no: AMP-D1). Primer sequences are in Table S2. Sanger sequencing was carried out by Source BioScience and analysed using Gap4 [3]. We sequenced cDNA from 6 *Whrn*$^{tm1b/tm1b}$ and 2 *Whrn*$^{+/tm1b}$ mice, 5 *Whrn*$^{wi/wi}$ and 1 *Whrn*$^{+/wi}$ mice, 3 *Whrn*$^{+/+}$ mice and 2 unrelated C57BL6/N wildtype mice as additional controls.

Western Blots

Retinas, inner ear sensory epithelia or brain tissue from each mouse was homogenized in 300 µl/100 mg tissue RadioImmune Precipitation Assay (RIPA) buffer, sonicated and boiled with Laemmli buffer for 10 min. The samples were separated by SDS polyacrylamide gel electrophoresis and transferred to a polyvinylidene fluoride (PVDF) membrane. The resulting PVDF membrane was sequentially subjected to blocking for 1 h, primary antibody (PB595) incubation overnight at 4°C and secondary antibody incubation for 1 h. The membrane was stripped prior to labelling with PB584. The protein bands were detected using ECL chemiluminescent substrate (Thermo Scientific Pierce).

Cochlear electrophysiology.

Detailed descriptions of these methods are described elsewhere [4]. In brief, mice were anaesthetized with urethane, 20 mg.g^{-1} intraperitoneal injections, the middle ear was opened and a Teflon-coated silver wire-recording electrode was placed on the round window, the ground at the vertex and reference on muscle behind the ear. A closed, calibrated sound delivery system was placed in the external ear canal. Cochlear microphonics were recorded using continuous tones stepped in frequency and intensity, recorded using a Brookdeal Lock-in amplifier. Summating potentials and compound action potentials were recorded using shaped tonebursts with rise/fall times of 1 ms, duration 15 ms, repetition rate 8.6 s^{-1}. A total of 28 whirler homozygotes plus 28 heterozygous littermate controls were analyzed with at least 6 in each group at ages P13, P15, P17 and P20.

Auditory Brainstem Electrophysiology (ABR).

Detailed descriptions of these methods are described in [5]. Mice were anaesthetised using an intraperitoneal injection of either urethane (2mg/g) or ketamine hydrochloride (100 mg.kg^{-1}, Ketaset, Fort Dodge Animal Health) and xylazine hydrochloride (10 mg/Kg, Rompun, Bayer Animal Health). Subcutaneous needle electrodes were placed over the right bulla (ground), left bulla (reference) and vertex (active). Electroencephalographic potentials were amplified, filtered (300 Hz-3 kHz) and averaged to produce the ABR. ABRs were recorded for calibrated tones (5ms duration, 1ms rise / fall time, 6-30 kHz) and clicks (10 µs duration) presented freefield at either 21.3 s^{-1} or 42.6 s^{-1} at a range of stimulus levels to determine threshold (dB SPL). Numbers of mice used: *Whrn*$^{wi/wi}$ P20 n=20, P56 n=2, P98 n=5; *Whrn*$^{+/wi}$ P20 n=16, P56 n=7, P98 n=5; *Whrn*$^{+/+}$ P20 n=4, P56 n=7. *Whrn*$^{tm1a/tm1a}$ P98 n=4, *Whrn*$^{+/tm1a}$ n=4, *Whrn*$^{+/+}$n=4. *Whrn*$^{tm1b/tm1b}$P28-35 n=8, P98 n=14; *Whrn*$^{+/tm1b}$ P28-35 n=4, P98 n=15; *Whrn*$^{+/+}$P28-35 n=9; P98 n=6.

ABR recordings were used to measure frequency tuning curves with a modified forward masking paradigm. A probe tone (12 kHz, 5ms duration, 1ms rise/fall time, presented at threshold +20dB) was presented with a 4ms gap after a masker tone of variable frequency, from 6 – 19.2 kHz in 9 steps, 10ms duration, with a 1ms rise/fall time, presented at levels ranging from 0-90dB SPL in 10dB steps. Masked threshold of the 12kHz probe tone was estimated for each masker frequency as the masker level that resulted in a 3dB (50% magnitude) reduction in ABR wave 1 amplitude modified from [6], and plotted as a function of masker frequency to produce an ABR frequency tuning curve for each mouse. Six *Whrn*$^{tm1b/tm1b}$ and 6 *Whrn*$^{+/tm1b}$ mice aged 14 weeks were used.

Distortion Product Otoacoustic Emission (DPOAE) measurements.

We measured distortion product otoacoustic emissions in *Whrn*^{tm1b} mice aged 14 weeks old, anaesthetised with Urethane, 0.1ml/10g bodyweight of a 20% solution, ip. We used Tucker Davis Technologies (TDT) BioSigRZ software, running on a TDT RZ6 auditory processor, driving a pair of TDT EC1 electrostatic transducers, with signals being recorded via an Etymotic ER-10B+ low noise DPOAE microphone. Stimuli were presented and signals recorded via a closed-field acoustic system sealed into the auditory meatus of the mouse. Stimulus tones

were presented in an F2:F1 ratio of 1.2. F2 tones were presented at 6, 12, 18, 24 & 30 kHz. F1 was presented at levels from 0-75dB in 5 dB steps. F2 was presented at 10 dB below the level of F1. The magnitude of the 2F1-F2 DPOAE component was extracted from a fast Fourier transform of the recorded microphone signal and plotted as a function of F2 level. For each F2 level, the 20 spectral line magnitudes surrounding the 2F1-F2 frequency were averaged to form a noise floor for each measurement. DPOAE threshold was defined as the lowest stimulus level where the emission magnitude exceeded 2 standard deviations above the mean noise floor. n=5 $Whrn^{tm1b/tm1b}$, 3 $Whrn^{+/tm1b}$.

Contact righting reflex

Adult mice were placed in a large glass petri dish, the lid positioned allowing the mouse to move but with contact of its feet and back with the glass, and the dish was turned over. The response of the mouse was observed and timed. Mice with normal balance turn over immediately, but mice with reduced balance function take longer to turn, presumably because the lack of vestibular input slows their ability to detect their position in relation to gravity. n=4 $Whrn^{tm1a/tm1a}$, 4 $Whrn^{+/tm1a}$, 4 +/+; 4 $Whrn^{tm1b/tm1b}$, 4 $Whrn^{+/tm1b}$, *4 +/+; 4* $Whrn^{wi/wi}$, 4 $Whrn^{+/wi}$

LacZ expression analysis

Inner ears (n= 1 heterozygote and 2 homozygotes at P5 and P28) were dissected out and fixed in fresh 4% paraformaldehyde for 45 minutes at 4^0C with rotation, decalcified in 10% EDTA before a detergent wash (2 mM $MgCl_2$; 0.02 % NP-40; 0.01 % sodium deoxycholate; in 0.1 M sodium phosphate buffer, pH 7.3 in PBS) for 30 min at room temperature. X-gal (Promega; cat.no. V394A) was added 1:50 to 500 µl of pre-warmed staining solution (5 mM $K_3Fe(CN)_6$; Ferrate (III) and 5 mM $K_4Fe(CN)_6$; Ferrate (II)), then ears were stained at 37°C in the dark for 1hr, washed in PBS and counterstained with Nuclear Fast Red (VWR, cat.no. 342094W). Cochlear and vestibular sensory epithelia were then microdissected, and whole mounts prepared for imaging with a Zeiss Axioskop 2.

FM1-43 uptake

For FM1-43 uptake, cochleae were processed as described in [7]. Briefly, cochleae from P10 mice (n=3) were excised and a small hole made at the apex of the bony cochlea. 1 ml of FM1-43FX (Invitrogen) at 5 μM in PBS at room temperature was perfused through the cochlear duct from oval and round windows for 30 seconds, and then washed 3X with 1 ml of PBS, followed by fixation with 4 % PFA for 20 min at room temperature. Organs of Corti were excised and mounted in ProLong Gold (Life Technologies) mounting media for imaging of the mid-apical region (60 – 70%) of the organ of Corti with a Zeiss Axioskop 2.

Immunofluorescence and stereocilia length quantification

Polyclonal antibodies against mouse WHRN (PB584 and PB595) were developed (Princeton Biomolecules and Covance) in rabbits immunized with a synthetic peptide (PB584: CDEETRKAREKERRRRLRRGA; PB595: SSLPQPHGSTLRQREDD). After affinity purification, specificity and selectivity was verified in COS7 cells transfected with WHRN-GFP (Figure S1). Rabbit polyclonal anti-EPS8 antibody (ab96144) was purchased from Abcam. Temporal bones were fixed by immersion in 4 % paraformaldehyde (PFA) in PBS; pH 7.4 for 20 min at room temperature. Sensory tissues were dissected in PBS, permeabilized with 0.5 % Triton X-100 for 30 min and blocked overnight at 4 °C with 4% bovine serum albumin in PBS. For labeling with PB595, tissue required treatment with 0.01M TRIS-EDTA (pH 9) for 45 minutes at 60 °C for antigen retrieval prior to blocking. Tissue was then incubated with primary antibody for 2 h, rinsed with PBS, stained with Alexa Fluor 488-, 568- or 405-conjugated secondary antibody (Life Technologies) for 1 h, counterstained with 0.001 U/µl Alexa Fluor 488- or 568-phalloidin (Molecular Probes), and mounted as whole sensory epithelia using Prolong Gold Antifade (Molecular Probes). Microscopy was performed using a Nikon TiE inverted fluorescence microscope, fitted with a spinning disc confocal head or an N-SIM Super Resolution System, 100x Apo TIRF 1.49 N.A. objective, and Andor Ixon camera. NIS-Elements imaging software was utilized for image acquisition. Stereocilia bundles were imaged at the mid-apical region, 60% - 70% from the basal end of the cochlea.

Stereocilia length measurements were made from confocal images, each of which contained several tens of stereocilia bundles within a single field of view and the same focal plane. Tissue was mounted by gently pressing against the coverslip to allow the bundles to virtually lay flat against the glass surface and remain within the ~500nm confocal optical section. The tallest stereocilia in each bundle was traced from base to tip, and this length was used. While the results may be a very slight underestimate of the length the relative measurements are internally consistent. Three animals of each genotype were used for the measurements.

Scanning Electron Microscopy (SEM)

The inner ear was gently flushed with ~0.3 ml of 2.5% glutaraldehyde fixative solution followed by two hours fixation at room temperature. Dissected cochlear and vestibular tissues were processed using an osmium-thiocarbohydrazide method [8], including three 1 hr incubations with 1% (w/v) OsO_4, alternated with two 1 hr incubations in 1% thiocarbohydrazide (w/v). The specimens were dehydrated, critical-point dried and viewed using a Hitachi S-4800 field emission scanning electron microscope. Stereocilia bundles were imaged at the mid-apical region, 60% - 70% from the basal end of the cochlea. N=3.

Supplemental References

1. White, J.K., et al., *Genome-wide generation and systematic phenotyping of knockout mice reveals new roles for many genes.* Cell, 2013. **154**(2): p. 452-64.
2. Skarnes, W.C., et al., *A conditional knockout resource for the genome-wide study of mouse gene function.* Nature, 2011. **474**(7351): p. 337-42.
3. Bonfield, J.K., K. Smith, and R. Staden, *A new DNA sequence assembly program.* Nucleic Acids Res, 1995. **23**(24): p. 4992-9.
4. Steel, K.P. and G.R. Bock, *Cochlear dysfunction in the jerker mouse.* Behav Neurosci, 1983. **97**(3): p. 381-91.
5. Ingham, N.J., S. Pearson, and K.P. Steel, *Using the Auditory Brainstem Response (ABR) to Determine Sensitivity of Hearing in Mutant Mice.* Curr Protoc Mouse Biol, 2011. **1**(2): p. 279-87.
6. Cheatham, M.A., et al., *Cochlear function in Prestin knockout mice.* J Physiol, 2004. **560**(Pt 3): p. 821-30.
7. Kurima, K., et al., *TMC1 and TMC2 Localize at the Site of Mechanotransduction in Mammalian Inner Ear Hair Cell Stereocilia.* Cell Rep, 2015. **12**(10): p. 1606-17.
8. Hunter-Duvar, I.M., *A technique for preparation of cochlear specimens for assessment with the scanning electron microscope.* Acta Otolaryngol Suppl, 1978. **351**: p. 3-23.
