# Supplementary material for: Alternative Splice Forms Influence Functions of Whirlin in Mechanosensory Hair Cell Stereocilia
Source: Cell Rep. 2016 Apr 21;15(5):935–43. doi: 10.1016/j.celrep.2016.03.081 (PMC4859837; doi:10.1016/j.celrep.2016.03.081)
Supplement: Document S2. Article plus Supplemental Information [file mmc2.pdf]

Cell Reports

Report

Alternative Splice Forms Influence Functions of Whirlin in Mechanosensory Hair Cell Stereocilia

Graphical Abstract

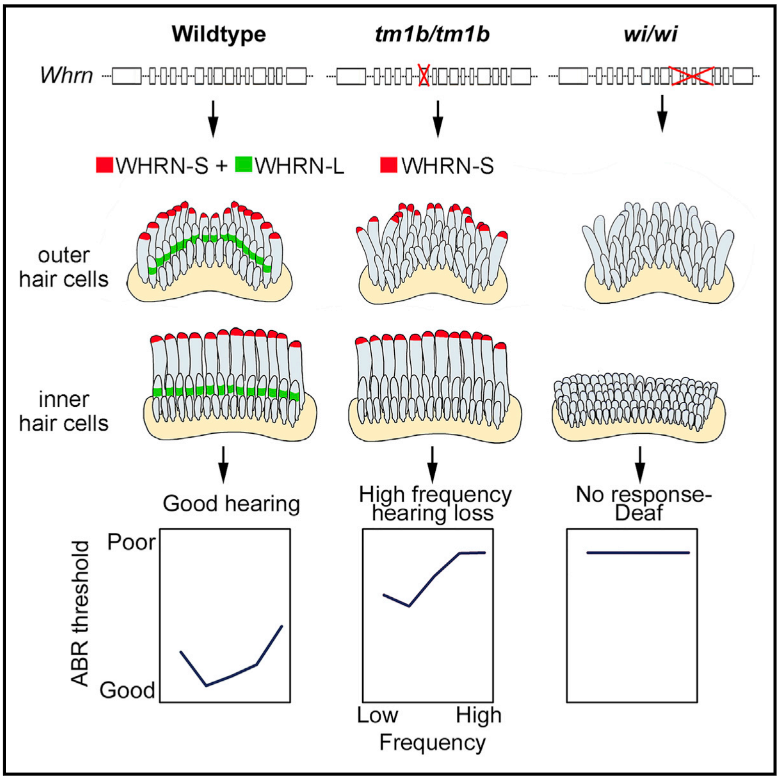

Authors

Seham Ebrahim, Neil J. Ingham, Morag A. Lewis, ..., Bechara Kachar, Johanna C. Pass, Karen P. Steel

Correspondence

karen.steel@kcl.ac.uk

In Brief

Ebrahim et al. show that two major isoforms of the *WHRN* gene have distinct localizations and functions within and across mechanosensory hair cells in the inner ear, and isoform-specific mutations are the likely cause of different auditory pathophysiologies associated with *WHRN* in mouse and humans.

Highlights

- Major WHRN isoforms WHRN-S and WHRN-L have distinct localizations within stereocilia
- Lack of WHRN-S and WHRN-L causes short stereocilia bundles and profound deafness
- In absence of WHRN-L, WHRN-S can preserve stereocilia length in certain hair cells
- Differential isoform expression underlies distinct phenotypes of known *Whrn* mutations

Accession Numbers

AY739121.1
NM_001008795.1

Ebrahim et al., 2016, Cell Reports *15*, 935–943
May 3, 2016 ©2016 The Authors
http://dx.doi.org/10.1016/j.celrep.2016.03.081

Cell Reports
Report

OPEN ACCESS
CellPress

Alternative Splice Forms Influence Functions of Whirlin in Mechanosensory Hair Cell Stereocilia

Seham Ebrahim,[1] Neil J. Ingham,[1,2] Morag A. Lewis,[1] Michael J.C. Rogers,[3] Runjia Cui,[4] Bechara Kachar,[4] Johanna C. Pass,[1,2] and Karen P. Steel[1,2,3,*]
[1]Wolfson Centre for Age-Related Diseases, King's College London, Guy's Campus, London SE1 1UL, UK
[2]Wellcome Trust Sanger Institute, Hinxton, Cambridge CB10 1SA, UK
[3]MRC Institute of Hearing Research, Nottingham NG7 2RD, UK
[4]National Institute on Deafness and Other Communications Disorders, NIH, Bethesda, MD 20892, USA
*Correspondence: karen.steel@kcl.ac.uk
http://dx.doi.org/10.1016/j.celrep.2016.03.081

SUMMARY

WHRN **(*DFNB31*) mutations cause diverse hearing disorders: profound deafness (DFNB31) or variable hearing loss in Usher syndrome type II. The known role of WHRN in stereocilia elongation does not explain these different pathophysiologies. Using spontaneous and targeted *Whrn* mutants, we show that the major long (WHRN-L) and short (WHRN-S) isoforms of WHRN have distinct localizations within stereocilia and also across hair cell types. Lack of both isoforms causes abnormally short stereocilia and profound deafness and vestibular dysfunction. WHRN-S expression, however, is sufficient to maintain stereocilia bundle morphology and function in a subset of hair cells, resulting in some auditory response and no overt vestibular dysfunction. WHRN-S interacts with EPS8, and both are required at stereocilia tips for normal length regulation. WHRN-L localizes midway along the shorter stereocilia, at the level of inter-stereociliary links. We propose that differential isoform expression underlies the variable auditory and vestibular phenotypes associated with *WHRN* mutations.**

INTRODUCTION

Inner ear sensory hair cells (HCs) transduce sound and head motion to electrical impulses via their mechanosensory hair bundles. Each hair bundle comprises dozens of specialized actin-filled protrusions, called stereocilia, organized in rows of graded heights. Stereocilia heights and organization have important effects on the HC's operating range, sensitivity, and frequency selectivity (Aranyosi and Freeman, 2004), and a number of proteins involved in regulating stereocilia morphology are essential for normal hearing (Dror and Avraham, 2009).

The PDZ domain-containing protein whirlin (WHRN) has been shown to localize to the tips of stereocilia, where it is involved in length-regulation (Delprat et al., 2005; Holme et al., 2002; Manor et al., 2011), and also to the stereocilia base, where it is thought to play a role in the bundle organization during development (Delprat et al., 2005). *Whrn* consists of 13 exons with two major splice variants: a long isoform (referred to here as WHRN-L) encoded by exons 1–13, composed of two PDZ domains at the N terminus followed by a proline-rich domain and a third PDZ at the C terminus; and a short form (WHRN-S), encoded by exons 6–13, which lacks PDZ1 and PDZ2 of the N terminus (Mburu et al., 2003). Mutations in *DFNB31* (encoding WHRN) cause profound non-syndromic deafness, DFNB31 (Mburu et al., 2003; Mustapha et al., 2002; Tlili et al., 2005). *DFNB31* has also been associated with Usher syndrome II, involving retinal degeneration and variable hearing loss (Aller et al., 2010; Audo et al., 2011; Ebermann et al., 2007). The underlying molecular pathogenic mechanisms are not known.

We addressed this outstanding question in the current study using the $Whrn^{wi/wi}$ mouse, in which both major WHRN isoforms are ablated (Mburu et al., 2003), and a mouse mutant, $Whrn^{tm1b(KOMP)Wtsi}$ (referred to here as $Whrn^{tm1b}$), which expresses WHRN-S but not WHRN-L. While the $Whrn^{wi/wi}$ mutant is profoundly deaf and exhibits circling and headbobbing behavior, we found that the $Whrn^{tm1b}$ mutant shows only moderate to severe hearing loss, suggesting that WHRN-S is sufficient to prevent complete loss of auditory function as well as vestibular dysfunction. We show that normal stereocilia are maintained in cochlear inner hair cells (IHCs) of the $Whrn^{tm1b}$ mutant, but outer hair cell (OHC) stereocilia morphology, organization, and function are affected. Similarly, while a subset of vestibular HCs in the $Whrn^{tm1b}$ mutant have abnormally short stereocilia, the remainder have stereocilia bundles with close-to-normal morphology. We use immunofluorescence with super-resolution, structured illumination microscopy (SIM) to determine the spatiotemporal localization of both WHRN isoforms along stereocilia. We show that WHRN-S localizes to the tips of stereocilia of IHCs from birth to adulthood, colocalizes with the actin regulatory protein EPS8, and is required for normal stereocilia length regulation. We propose that the localization of WHRN-L along the stereocilia shaft coincides with inter-stereociliary links, such as lateral links or horizontal top connectors, and its absence leads to disorganized bundles. Thus, WHRN isoforms are expressed differentially across HC types and within stereocilia, where they play distinct roles in organization and elongation.

This is an open access article under the CC BY license (http://creativecommons.org/licenses/by/4.0/).

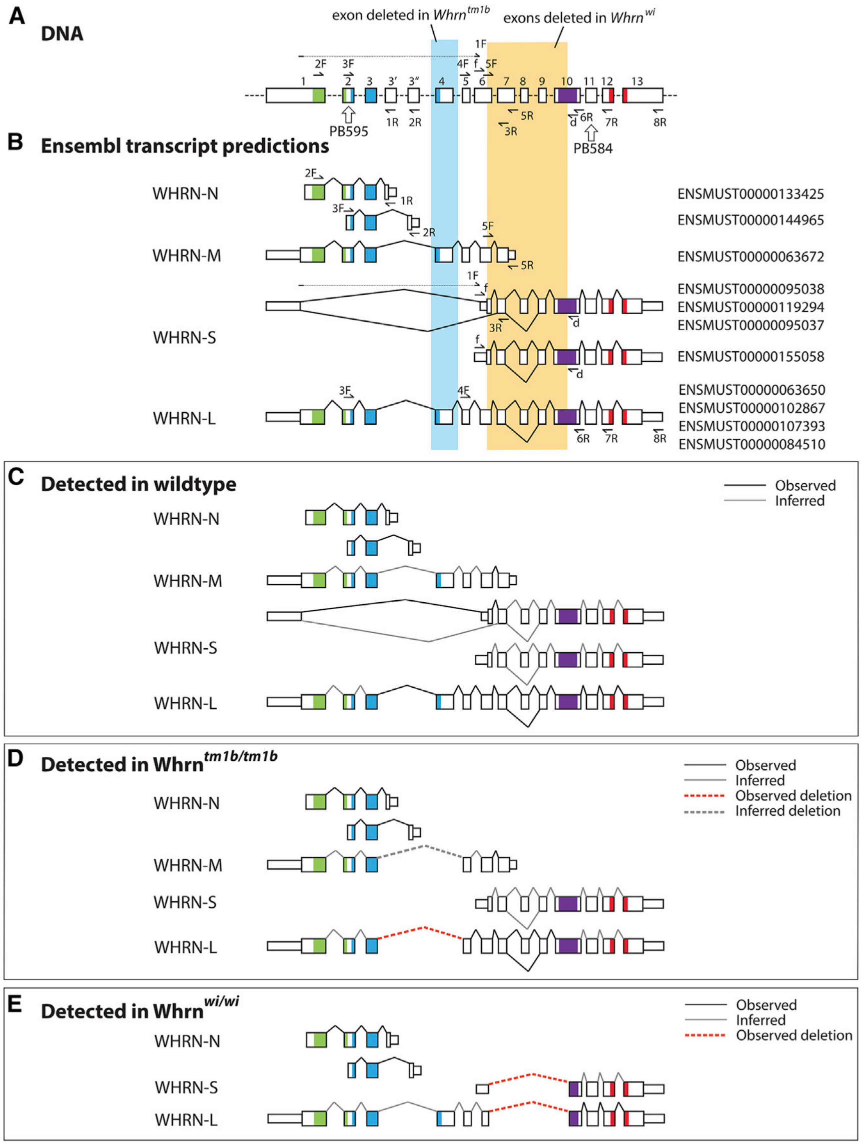

Figure 1. *Whrn* Isoform Expression

(A) Diagram of *Whrn* exons showing deletions in *Whrn*$^{wi/wi}$ (orange) and *Whrn*$^{tm1b/tm1b}$ (blue), with regions coding for WHRN domains colored (green, PDZ1; blue, PDZ2; red, PDZ3; purple, proline-rich domain). Vertical arrows mark target region of antibodies PB584 and PB595. Horizontal arrows mark locations of primers.

(B) Ensembl predictions of *Whrn* isoforms. Narrow boxes indicate UTRs and wider boxes protein-coding regions. Left: isoform names used in this paper. Right: Ensembl transcript IDs. ENSMUST00000155058 is classified as a retained intron transcript but was recently found in the inner ear (Mathur et al., 2015b) and results in a protein sequence identical to transcript ENSMUST00000119294, one of the WHRN-S isoforms, when translated. (GenBank accession numbers provided in Table S1). Primers used to detect each isoform are shown on the relevant isoform.

(C–E) Transcripts detected in wild-type (C), *Whrn*$^{tm1b/tm1b}$, (D) and *Whrn*$^{wi/wi}$, and (E) inner ears. Black lines indicate splice junctions observed by sequencing. Grey lines indicate splice junctions inferred by presence of the isoform, but not observed by sequencing.

See also Figure S1.

RESULTS

Whrn$^{wi/wi}$ and *Whrn*$^{tm1b/tm1b}$ Mice Express Different WHRN Isoforms

In this study, we analyzed the whirler (*Whrn*$^{wi/wi}$) mouse (Mburu et al., 2003; Mogensen et al., 2007), which has a spontaneous deletion encompassing the majority of exons 6–10 of the *Whrn* gene, resulting in the ablation of both major isoforms of WHRN, WHRN-L and WHRN-S (Figures 1A and 1B). We also used a mutant, *Whrn*$^{tm1b(KOMP)Wtsi}$ (referred to from here as *Whrn*^{tm1b}), in which exon 4 of the *Whrn* gene is deleted and a cassette including a β-galactosidase reporter (lacZ), is inserted into intron 3 (Figures S1A and S1B).

Ensembl predicts five main WHRN isoforms: two short N-terminal isoforms (here referred to collectively as WHRN-N), a midsize isoform (WHRN-M), and several splice variants of both the long isoform (WHRN-L) and the short C-terminal isoform (WHRN-S) (Figure 1B). We examined the transcripts present in inner ear tissue of each mutant by reverse transcriptase (RT) PCR followed by capillary sequencing (Figure 1B).

In the wild-type inner ear, we found both WHRN-N isoforms, WHRN-M, WHRN-L, and two variants of WHRN-S (Figure 1C). The two WHRN-N isoforms were also found in both *Whrn*^{wi} and *Whrn*^{tm1b} homozygotes (Figures 1D and 1E). We were unable to test for the presence of WHRN-M in *Whrn*^{wi} homozygotes because the only specific primer set fell in the deletion; if this isoform is transcribed, it is predicted to be truncated. We did confirm the presence of WHRN-M in *Whrn*^{tm1b} homozygotes but could not observe the lack of exon 4 in this isoform because exon 4 was not part of the sequence amplified by the specific primer set (Figure 1D).

WHRN-L was detected in both *Whrn*^{wi} and *Whrn*^{tm1b} homozygotes (Figures 1D and 1E), and the deletion in each mutant allele was observed by sequencing. In the *Whrn*^{wi} sequence, there is a clean break from exon 6 to exon 10, which introduces a frameshift. The *Whrn*^{tm1b} sequence lacks exon 4 but has a section of 115 bp between exon 3 and exon 5 that is an exact match to the region around the splice junction of the second exon of *En2*. It is likely that this is because the trapping cassette used in making the original tm1a allele includes the mouse *En2* splice acceptor (Skarnes et al., 2011). If either the *Whrn*^{wi} or the *Whrn*^{tm1b} WHRN-L transcripts are translated, the mutations are

OPEN ACCESS
CellPress

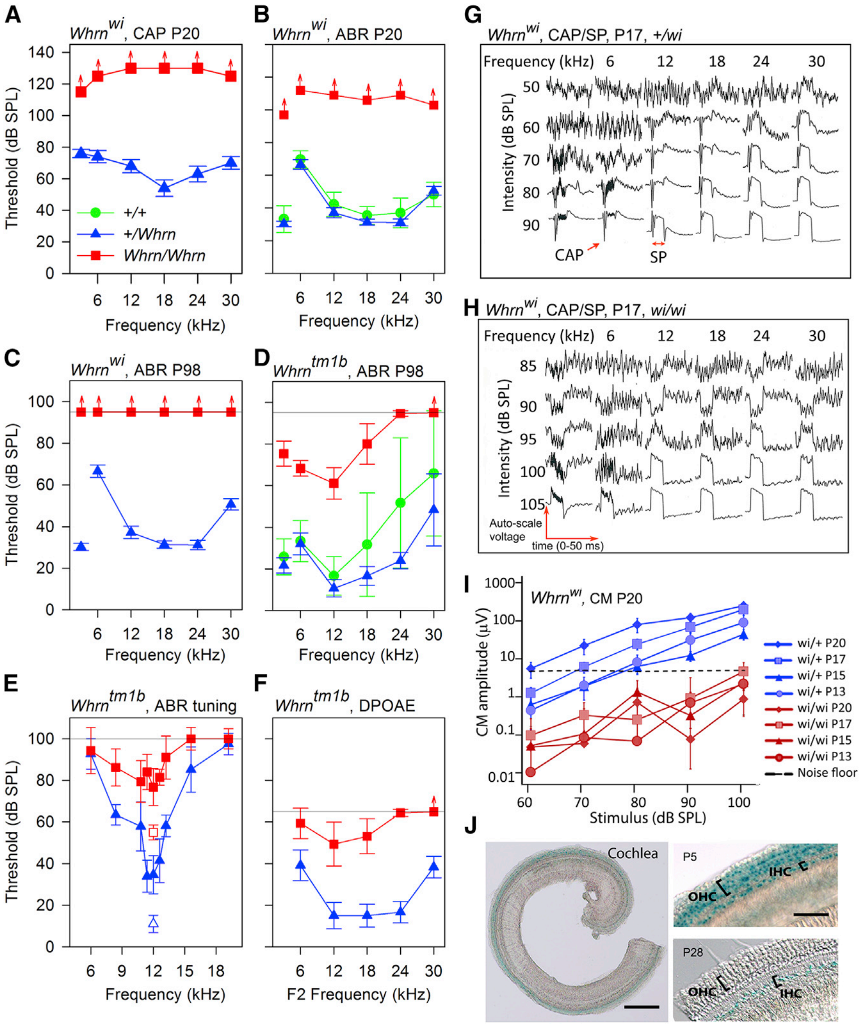

Figure 2. Auditory Electrophysiology and *Whrn* Expression in *Whrn*$^{wi/wi}$ and *Whrn*$^{tm1b/tm1b}$ Mice

(A) Mean CAP threshold (±SD) in P20 *Whrn*^{wi} mice.
(B) Mean ABR threshold (±SD) in P20 *Whrn*^{wi} mice.
(C) Mean ABR threshold (±SD) in P98 *Whrn*^{wi} mice.
(D) Mean ABR threshold (±SD) in P98 *Whrn*^{tm1b} mice.
(E) Mean ABR masked tuning curves (±SD) in P98 *Whrn*^{tm1b} mice. Open symbols indicate mean (±SD) 12 kHz probe tone threshold. Filled symbols indicate mean (±SD) masked tuning thresholds.
(F) Mean 2F1-F2 DPOAE threshold (±SD) in P98 *Whrn*^{tm1b} mice. Red arrows on symbols indicate maximum sound pressure level tested.
(G) Waveforms from *Whrn*$^{+/wi}$ mouse (P17) showing range of responses. CAP and SP responses are indicated. SP can be either negative (e.g., 12 kHz at 70 dB SPL) or positive (high frequencies, high intensities).
(H) Waveforms from a *Whrn*$^{wi/wi}$ mutant (P17), showing positive and negative SPs but no CAP.
(I) Cochlear microphonic amplitudes plotted as a function of stimulus intensity for a 6 kHz tone.
(J) LacZ staining in organ of Corti from *Whrn*$^{tm1b/tm1b}$ mice at P5 (left: whole cochlea, right top: magnified) and P28 (bottom right).
See also Figure S2.

predicted to introduce a stop codon and result in a truncated protein.

WHRN-S was also detected in both *Whrn*^{wi} and *Whrn*^{tm1b} homozygotes (Figures 1D and 1E). The *Whrn*^{tm1b} product was identical to the wild-type product. The deletion in the *Whrn*^{wi} allele was clear, just as observed in the WHRN-L transcript. In the WHRN-S isoform, the *Whrn*^{wi} deletion starts in the 5′UTR and removes the start codon, so it is unlikely that any protein is produced from the mutant transcript.

Whrn$^{wi/wi}$ and *Whrn*$^{tm1b/tm1b}$ Mice Show Distinct Cochlear Physiology, Vestibular Phenotypes, and Whrn Expression Pattern

Phenotypic differences between the two mutants were immediately apparent: *Whrn*$^{wi/wi}$ mice are profoundly deaf and exhibit headbobbing and circling behavior characteristic of severe vestibular dysfunction, while *Whrn*$^{tm1b/tm1b}$ mice intriguingly showed no overt vestibular abnormalities. We tested the contact righting reflex, which uses a combination of visual, vestibular, and somatosensory inputs to make postural adjustments after displacement, in adult mice. When *Whrn*$^{tm1b/tm1b}$ mice were placed on their backs, they immediately turned over to rest on all four feet, while *Whrn*$^{wi/wi}$ mice took several seconds to right themselves.

We next evaluated cochlear physiology. In *Whrn*$^{wi/wi}$ mice, recordings from the round window showed no compound action potentials (CAPs) even at the highest stimulus intensities used (Figure 2A). *Whrn*$^{wi/wi}$ mutants also showed no auditory brainstem response (ABR) to the highest sound stimulus at P20 and P98 (Figures 2B and 2C). *Whrn*$^{+/wi}$ mice had comparable ABR thresholds to *Whrn*$^{+/+}$ mice, suggesting the mutation was completely recessive (Figure 2B). In contrast, 14-week-old mice homozygous for the *Whrn*$^{tm1b/tm1b}$ allele showed a profound impairment only at higher frequencies (24–30 kHz) and only moderate impairment at lower frequencies (Figure 2D). Hearing loss in *Whrn*$^{tm1b/tm1b}$ mice did not progress between 4 and 14 weeks (data not shown).

We assessed frequency tuning in *Whrn*^{tm1b} mice by measuring forward masked frequency tuning curves using a 12 kHz probe tone presented at 20 dB above threshold (Figure 2E). *Whrn*$^{+/tm1b}$ mice produced a mean tuning curve with a sensitive tip located close to the 12 kHz probe tone. In contrast, *Whrn*$^{tm1b/tm1b}$ mutants produced a mean tuning curve that was flat across frequencies (Figure 2E), suggesting impaired function of OHCs. OHC dysfunction in *Whrn*$^{tm1b/tm1b}$ mutants was further indicated by measurements of 2F1-F2 distortion product otoacoustic emission (DPOAE) thresholds (Figure 2F). *Whrn*$^{+/tm1b}$ mice had sensitive DPOAE thresholds for F2 frequencies, reflecting the shape of the ABR audiogram; *Whrn*$^{tm1b/tm1b}$ mutants displayed moderate

elevations in DPOAE threshold for F2 frequencies of 6–18 kHz, while DPOAEs from higher F2 frequencies were often not evoked even at the highest stimulus levels tested (Figure 2F).

HCs of *Whrn*$^{wi/wi}$ mutant mice degenerate from around P21 (Holme et al., 2002), so we analyzed mice younger than this age to investigate HC function before the onset of degeneration. We recorded cochlear microphonics (an alternating voltage with the same frequency as the stimulus) that reflect OHC function and found that responses from *Whrn*$^{wi/wi}$ mutants aged P13 to P20 were below detection limits, within the noise floor (Figure 2I). We also recorded summating potentials (Figures 2G and 2H), which are a sustained shift in potential for the duration of the 15-ms tone burst, and represent asymmetry of the receptor current between positive and negative phases of the acoustic stimulus (Dallos et al., 1972; Harvey and Steel, 1992). Summating potentials were detected in 27 of the 28 *Whrn*$^{wi/wi}$ mutants studied, albeit at high stimulus intensities, suggesting that some HCs can depolarize in response to sound (example traces in Figure 2H). These data are consistent with previous reports where mechanotransduction currents were recorded from early postnatal HCs in *Whrn*$^{wi/wi}$ mutants (Stepanyan et al., 2006).

Finally, we used lacZ staining to determine the endogenous expression pattern of WHRN-L in the inner ear. Since the lacZ reporter within the *Whrn*^{tm1b} allele is in the third intron (Figure S1A), it will be transcribed wherever WHRN-L is transcribed. The coding sequence of WHRN-S begins partway through exon 6, so the lacZ will not report WHRN-S expression. At P5, when stereocilia bundles are still developing, lacZ expression was detected in both IHCs and OHCs of the organ of Corti (Figure 2I). In the vestibular organs lacZ expression was very strong in a subpopulation of HCs: the extrastriolar region of the utricular macula (Figures S2A and S2C) and the peripheral zone of the crista ampularis (Figures S2B and S2D). Conversely, lacZ expression was not detected in the striolar and central zones (Figure S2, arrowheads). At P28, when HCs are mature, the pattern of lacZ expression in the vestibular organs remained consistent (Figure S2), but lacZ was no longer detected in OHCs of the organ of Corti (Figure 2I). These data suggest that endogenous expression of WHRN-L is high in cochlear OHCs in the early postnatal period but declines after functional maturity is reached.

WHRN-S Expression Is Necessary and Sufficient for Normal IHC Stereocilia Length, but Both WHRN-S and WHRN-L Are Required for Normal OHC Stereocilia Bundle Morphology

To determine whether the observed differences in WHRN isoform expression between the *Whrn*$^{wi/wi}$ and *Whrn*$^{tm1b/tm1b}$ mice led to differences in stereocilia bundle morphology, we used scanning electron microscopy (SEM) and confocal fluorescence microscopy to image stereocilia bundles in the organ of Corti. We found, as previously reported (Holme et al., 2002; Mburu et al., 2003; Mogensen et al., 2007), that IHC stereocilia in the *Whrn*$^{wi/wi}$ mutant were abnormally short (Figures 3F and 3I) and OHC stereocilia bundles were rounded (“U” rather than “W” shaped) and had irregular spacing between stereocilia rows (Figure 3E) compared to littermate controls (Figures 3A and 3B). *Whrn*$^{tm1b/tm1b}$ OHC stereocilia bundles had a similar phenotype to the *Whrn*$^{wi/wi}$ mutants (Figure 3C). Strikingly, however, the morphology of *Whrn*$^{tm1b/tm1b}$ IHC stereocilia bundles (Figures 3D and 3H) appeared normal (Figures 3B and 3G). We measured the lengths of stereocilia from IHCs and OHCs from wild-type, *Whrn*$^{tm1b/tm1b}$, and *Whrn*$^{wi/wi}$ mutants and found no significant difference between wild-type and *Whrn*$^{tm1b/tm1b}$ mice (Figure 3J). This suggests that the expression of WHRN-S is necessary to maintain normal IHC stereocilia length at P21. Looking for interstereocilia links, we found lateral links between adjacent stereocilia in some *Whrn*$^{tm1b/tm1b}$ OHC bundles (Figure S3). This was also consistent with lateral links that have been previously reported in OHC stereocilia of *Whrn*$^{wi/wi}$ mice (Mogensen et al., 2007).

We next assessed uptake of FM1-43, a compound that permeates the stereocilia mechanotransducer channels (Gale et al., 2001), in cochlear HCs from *Whrn*$^{tm1b/tm1b}$ and *Whrn*$^{wi/wi}$ mice at P10 to determine whether their abnormal morphology affected mechanoelectrical transduction (MET). Uptake of FM1-43 was observed in both IHCs and OHCs of both *Whrn*$^{tm1b/tm1b}$ and *Whrn*$^{wi/wi}$ mice (Figures S3H and S3I). OHCs load more dye than IHCs, as previously reported (Gale et al., 2001). These data are consistent with our finding of summating potential responses in the *Whrn*^{wi} mutants and previous reports of MET recordings from *Whrn*^{wi} mutant OHCs (Stepanyan et al., 2006), suggesting that some transduction can occur in *Whrn* mutant HCs at high stimulus levels.

Vestibular Stereocilia Bundles Show Distinct Morphologies in *Whrn*$^{wi/wi}$ and *Whrn*$^{tm1b/tm1b}$ Mice

We analyzed stereocilia in the vestibular organs in each mutant line to determine whether bundle morphology was responsible for the observed differences in vestibular phenotype between them. In *Whrn*$^{wi/wi}$ mutants stereocilia were consistently markedly shorter (Figures 3O, 3P, 3U, 3V, S3C, and S3F) than in controls (Figures 3K, 3L, 3Q, 3R, S3A, and S3D), across all HCs. In the *Whrn*$^{tm1b/tm1b}$ mice, however, we observed interesting regional differences in bundle morphology: while stereocilia bundles in the extrastriolar region of the utricular and saccular maculae were short (Figures 3M and 3S) as in the *Whrn*$^{wi/wi}$ mutants (Figures 3O and 3U), bundles in the striolar region were more variable, with longer stereocilia and a closer to normal staircase organization (Figures 3N and 3T). We quantified this by measuring the length of tallest stereocilia in extrastriolar (Figure 3W) and striolar (Figure 3X) bundles in *Whrn*$^{+/tm1b}$, *Whrn*$^{tm1b/tm1b}$, and *Whrn*$^{wi/wi}$ mice. In both *Whrn*$^{tm1b/tm1b}$ and *Whrn*$^{wi/wi}$ mutants, the tallest stereocilia in extrastriolar bundles showed an ~80% reduction in length compared to *Whrn*$^{+/tm1b}$ mice (Figure 3W). In the striolar region, the tallest stereocilia in *Whrn*$^{wi/wi}$ mutants again showed an ~80% reduction in length compared to *Whrn*$^{+/tm1b}$ mice, but in *Whrn*$^{tm1b/tm1b}$ mutants the length reduction was closer to ~50% (Figure 3X). The regional differences in stereocilia morphology were particularly pronounced in the crista ampularis of the *Whrn*$^{tm1b/tm1b}$ mutants (Figures S3A–S3F), where bundles in the central regions were much shorter (Figure S3B) than heterozygous controls (Figure S3A), but bundles in the peripheral zone (Figure S3E) had morphology, comparable to control bundles (Figure S3D). These data are consistent with a similar phenotype reported in *Whrn*$^{neo/neo}$ mice (Mathur et al., 2015a). Because the stereocilia in the crista ampularis can be up to 100 μm long, it was not

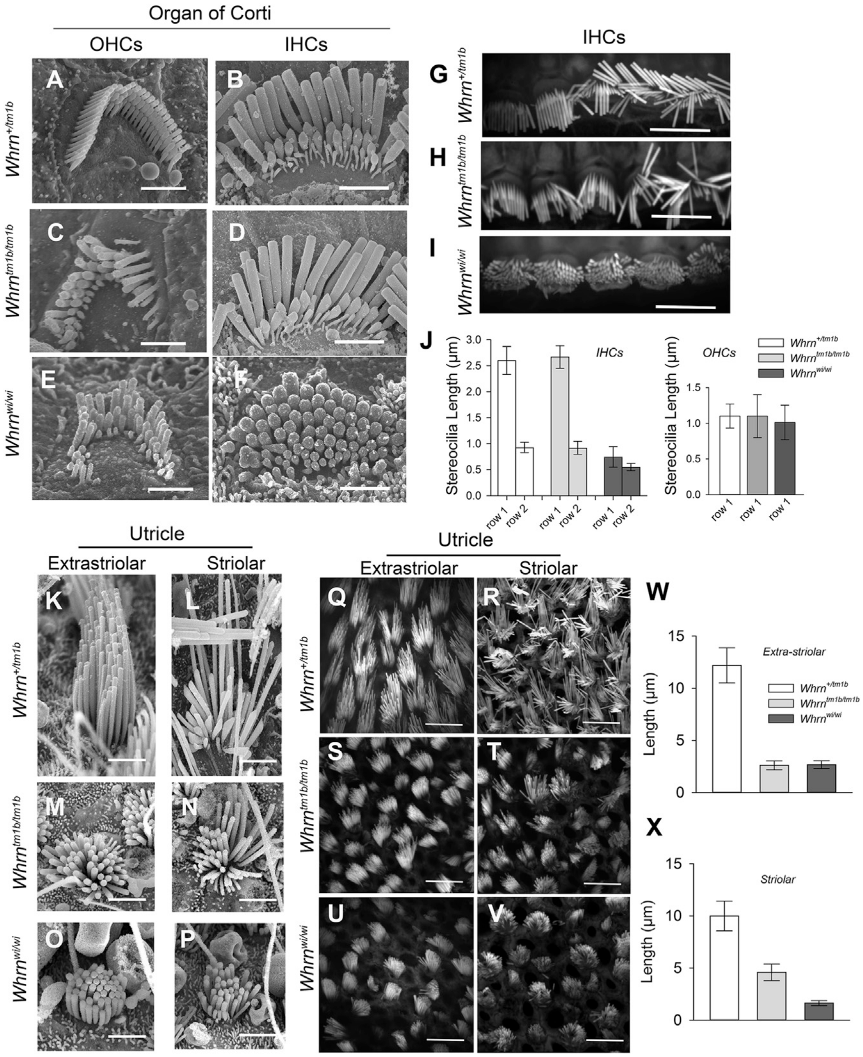

Figure 3. Differential *Whrn* Isoform Expression Affects Stereocilia Bundle Morphology

(A and B) Scanning electron microscopy (SEM) images of OHCs (A) and IHCs (B) from P21 *Whrn*$^{+/tm1b}$ mice. Scale bars, 2 µm.

(C and D) SEM images of OHCs (C) and IHCs (D) from P21 *Whrn*$^{tm1b/tm1b}$ mice. Scale bars, 2 µm.

(E and F) SEM images of OHCs (E) and IHCs (F) from P21*Whrn*$^{wi/wi}$ mice. Scale bars, 2 µm.

(G–I) Confocal fluorescence images of IHC stereocilia (labeled with phalloidin) from P10 *Whrn*$^{+/tm1b}$ (G), *Whrn*$^{tm1b/tm1b}$ (H), and *Whrn*$^{wi/wi}$ (I) mice. Scale bars, 5 µm.

(J) Stereocilia length (±SD) from the tallest (row 1) and middle (row 2) row of IHC bundles and the tallest row (row 1) of OHCs of *Whrn*$^{+/tm1b}$, *Whrn*$^{tm1b/tm1b}$, and *Whrn*$^{wi/wi}$ mice.

(K–P) SEM images of vestibular stereocilia bundles in the extrastriolar and striolar region of the utricle of P21 *Whrn*$^{+/tm1b}$ (K and L), *Whrn*$^{tm1b/tm1b}$ (M and N), and *Whrn*$^{wi/wi}$ (O and P) mice. Scale bars, 2 µm.

(Q–V) Confocal fluorescence images of vestibular stereocilia bundles in the extrastriolar and striolar region of the utricle in P10 *Whrn*$^{+/tm1b}$ (Q and R), *Whrn*$^{tm1b/tm1b}$ (S and T), and *Whrn*$^{wi/wi}$ (U and V) mice. Scale bars, 5 µm.

(W and X) Length of the tallest stereocilia (±SD) from extrastriolar (W) and striolar (X) vestibular bundles.

See also Figure S3.

possible to measure the precise lengths of stereocilia in this organ. However, the presence of regions in all vestibular organs of HCs with stereocilia morphology that was either normal or closer-to-normal (compared with *Whrn*$^{wi/wi}$ mutants), likely explains the lack of overt vestibular phenotype in the *Whrn*$^{tm1b/tm1b}$ mutants.

WHRN-S and WHRN-L Have Distinct Localizations within Stereocilia in Auditory Hair Cells

To explore the molecular mechanisms underlying the differences in stereocilia length between *Whrn*$^{tm1b/tm1b}$ and *Whrn*$^{wi/wi}$ mutants, we investigated the precise localization of WHRN-S and WHRN-L within stereocilia. We used a custom antibody (PB584) against an epitope just upstream of the PDZ3 domain (Figure 1A), which labels both WHRN-L and WHRN-S (Figure S1). Therefore, both isoforms should be labeled in stereocilia of control mice, only WHRN-S in *Whrn*$^{tm1b/tm1b}$ mice, and no immunoreactivity is expected in the *Whrn*$^{wi/wi}$ mutants where both isoforms are ablated. We labeled all three mouse lines at P10 and found that in control mice, consistent with previous reports, WHRN localized at stereocilia tips of the tallest row in IHCs (Figures 4A and 4C, white arrowhead). We also detected a distinct distribution of WHRN, proximal to the tips of shorter stereocilia (Figures 4A–4C, yellow arrowhead). In OHCs at P10, the localization of WHRN at the stereocilia tips of the tallest row was not detected (some localization at the tips of OHCs was detected at P8; see Figures S4D and S4E), while its localization proximal to the tips of shorter stereocilia persisted (Figures 4D and 4E). To pinpoint this latter localization in IHC (Figures S4A–S4C) and OHC stereocilia of wild-type mice (Figures S4D–S4F), we used high-resolution spinning disc confocal microscopy (Figures S4A and S4D) as well as SIM (Figures S4B–S4F). We used the SIM images to track this localization of WHRN relative the tip and base of the second row of stereocilia (Figure S4G) and found localization within the upper half of the shorter stereocilia. Interestingly, in *Whrn*$^{tm1b/tm1b}$ mice, which express WHRN-S but not WHRN-L, PB584 labeled the tips of the tallest row of stereocilia in IHCs (Figures 4F and 4H, white arrowhead), but localization in shorter stereocilia was no longer detectable (Figures 4F–4H). The absence of WHRN labeling in the shorter row, which was also noted in OHCs (Figures 4I and 4J), suggests that this particular localization can be attributed to WHRN-L. The localization of WHRN-L proximal to the tips of the second row of stereocilia was further verified (Figure S4H) using an isoform-specific antibody PB595, (validated in Figure S1), directed to the N terminus of WHRN-L (Figure 1A). Antigen retrieval was needed for stereocilia labeling with PB595,

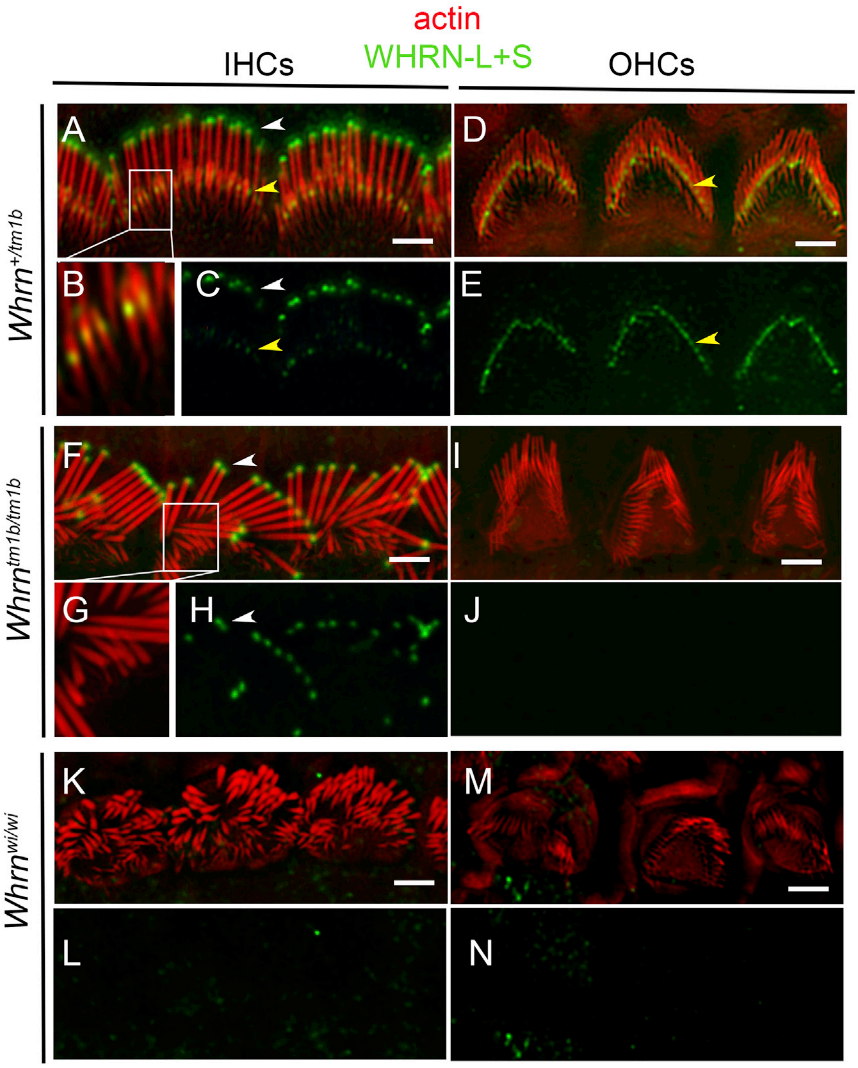

Figure 4. *Whrn* Isoforms Show Distinct Localizations within Stereocilia

(A–C) WHRN (green) localizes in IHC stereocilia (red) to two distinct regions in P10 $Whrn^{+/tm1b}$ mice: stereocilia tips of the tallest row (white arrowhead) and proximal to the tips of the shorter row of stereocilia (yellow arrowhead). Boxed region in (A) is shown in high-magnification (B).

(D and E) WHRN (green) localizes in OHC stereocilia (red) predominantly to the shorter row of stereocilia in P10 $Whrn^{+/tm1b}$ mice.

(F–H) WHRN (green) localizes in IHC stereocilia (red) to stereocilia tips of the tallest row (F, arrowhead) in $Whrn^{tm1b/tm1b}$ mice. WHRN localization to shorter stereocilia is not detected. Boxed region magnified in (G).

(I and J) WHRN (green) localization is not detected in OHC stereocilia (red) in $Whrn^{tm1b/tm1b}$ mice (I).

(K–N) WHRN label in $Whrn^{wi/wi}$ mutant stereocilia. WHRN (green) is not detected in $Whrn^{wi/wi}$ stereocilia (red) from IHCs (K and L) or OHCs (M and N). Scale bars, 2 μm.

See also Figure S4.

suggesting that the epitope (between PDZ1 and PDZ2; Figure 1A) may be sterically challenging to access. Despite higher background fluorescence as a result of antigen retrieval, no stereocilia tip localization was detected with PB595 (Figure S4H, white arrows). No PB595 immunoreactivity was detected in tm1b/tm1b mice (Figures S4I and S4J). No WHRN immunoreactivity was detected in the IHCs or OHCs of $Whrn^{wi/wi}$ mutants, in which both WHRN-S and WHRN-L are ablated (Figures 4K–4N). Together, these data indicate that WHRN-S and WHRN-L have distinct localizations within stereocilia.

WHRN-S Colocalizes with EPS8 and Both Are Required for Normal Stereocilia Length

EPS8 is an actin regulatory protein that has been shown to be transported, along with WHRN, by MYO15a to stereocilia tips, where it has been implicated in stereocilia elongation (Manor et al., 2011). To investigate the specific roles of WHRN-S and WHRN-L, we asked whether the distribution of EPS8 varied in stereocilia from $Whrn^{wi/wi}$ and $Whrn^{tm1b/tm1b}$ mice. In $Whrn^{tm1b/tm1b}$ mice, EPS8 localized to the tallest row of stereocilia in IHCs (Figures 5A and 5B) and OHCs (Figure 5D) and colocalized strongly with WHRN-S (Figures 5A–5C), while no WHRN-L was detected. In $Whrn^{wi/wi}$ mice, EPS8 localization to stereocilia tips at least partially persisted in both IHCs (Figure 5E, arrowheads) and OHCs (Figure 5F, arrowheads), consistent with previous reports (Manor et al., 2011). Strikingly, however, in *Eps8* null mutants (Offenhäuser et al., 2006) that also have short stereocilia (Manor et al., 2011), we found that WHRN-S immunofluorescence was not detected at the tips of the tallest row of stereocilia in IHCs (Figure 5G) or OHCs (Figure 5H).WHRN-L localization proximal to the tips of the shorter rows was maintained in OHCs (Figure 5H), but not detected in IHCs (Figure 5G). Together, these data show that in the absence of EPS8, WHRN-S is not able to properly localize to stereocilia tips. Furthermore, that $Whrn^{wi/wi}$ mice have short stereocilia despite the presence of EPS8 at stereocilia tips suggests that in HCs, both EPS8 and WHRN-S are required to modulate stereocilia elongation.

DISCUSSION

In the current study, we show that the major WHRN isoforms, WHRN-S and WHRN-L, show differential expression within stereocilia and across HC type. While lack of both isoforms results in abnormally short stereocilia across HCs, we find that WHRN-S alone is sufficient to maintain normal stereocilia bundle morphology in IHCs and a subset of vestibular HCs. Accordingly, mice expressing WHRN-S have a milder auditory phenotype than mice lacking both WHRN-S and WHRN-L

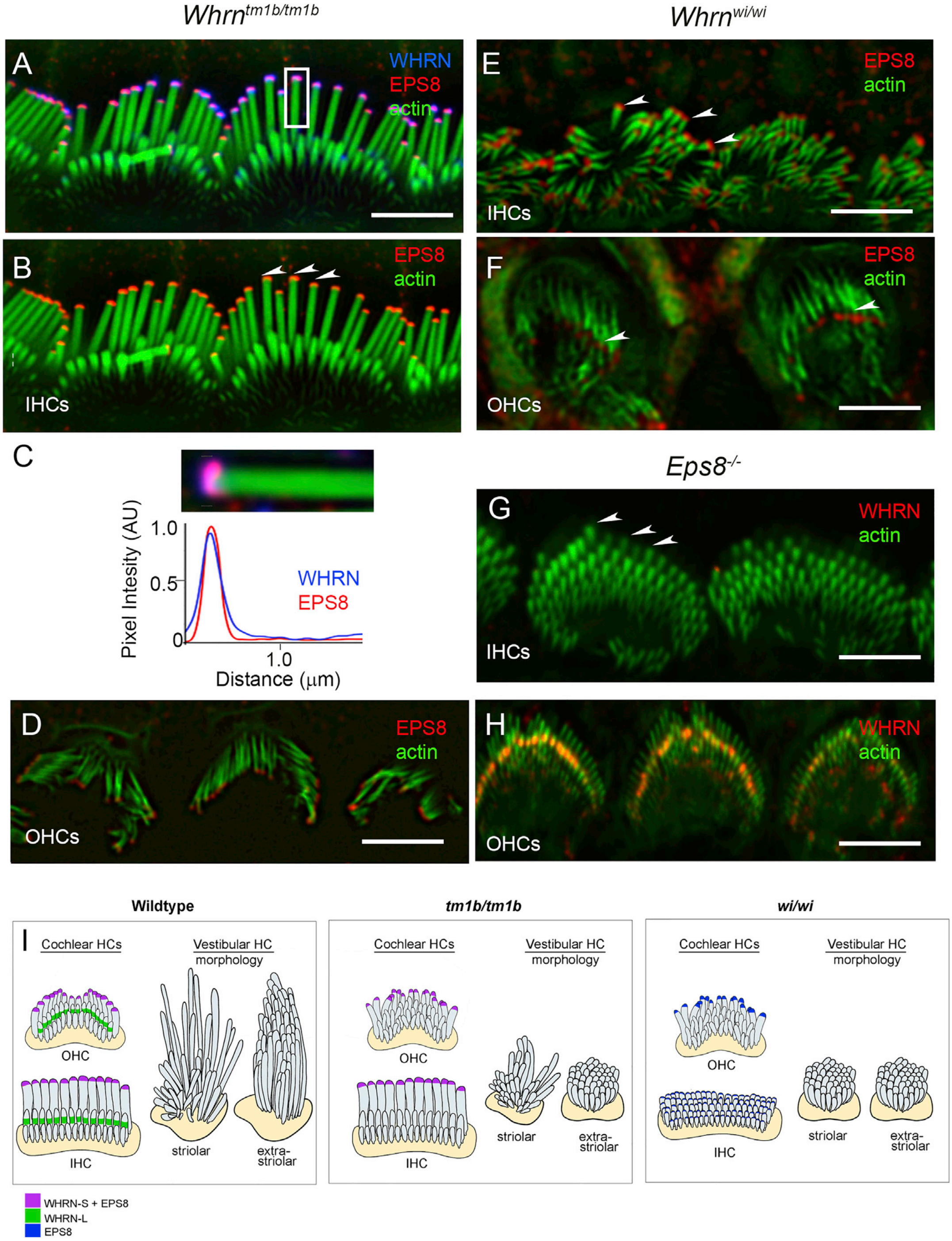

Figure 5. WHRN-S Localization at Stereocilia Tips Overlaps with EPS8 and Both Proteins Are Required for Normal Stereocilia Elongation

(A and B) Localization of WHRN and EPS8 to stereocilia tips. SIM image of IHC stereocilia (green) from P11 *Whrn*$^{tm1b/tm1b}$ mice. WHRN-S (blue) and EPS8 (red) colocalize at stereocilia tips (arrowheads).

(C) High mag of stereocilium from box in (A) and corresponding fluorescence intensity profile (below).

(D) EPS8 (red) in OHC stereocilia (green) from P11 *Whrn*$^{tm1b/tm1b}$ mice.

(E and F) EPS8 (red) immunofluorescence in stereocilia (green) from P10 *Whrn*$^{wi/wi}$ mutants shows stereocilia tip localization (arrowheads) in IHCs (E) and OHCs (F).

(G and H) WHRN (red) immunofluorescence in stereocilia (green) from P6 *Eps8* null mouse. No stereocilia tip localization observed in IHCs (G) or OHCs (H). WHRN-L labeling is still detected in OHCs (H).

(I) Schematic summarizing WHRN isoform and EPS8 localization in stereocilia of cochlear hair cells, and vestibular stereocilia bundle morphology in wild-type, *Whrn*$^{tm1b/tm1b}$, and *Whrn*$^{wi/wi}$ mice. Scale bars, 2 μm.

(Holme et al., 2002) and also show no overt vestibular problems. These findings are consistent with mapped pathogenic variants in human patients and their respective phenotypes: DFNB31 patients, who suffer from profound prelingual sensorineural hearing loss (Mustapha et al., 2002), have mutations within exons 10 and 11 of *DFNB31* (Mburu et al., 2003; Mustapha et al., 2002; Tlili et al., 2005), which affect the PDZ3 domain of both WHRN-L and WHRN-S, likely resulting in truncated, non-functional proteins. Mutations in patients with USH2D, who have milder and more variable hearing abnormalities, have been localized to exons 1 and 2 and intron 2 of *DFNB31* (Audo et al., 2011; Besnard et al., 2012; Ebermann et al., 2007), which affect PDZ1 and PDZ2 of WHRN-L, but likely have no impact on the expression of WHRN-S. Our findings are also consistent with the report of a different mutation targeting exon 1 of *Whrn* in the mouse, where IHCs appeared normal and OHCs showed similar abnormalities as we report here (Yang et al., 2010). While genetic analysis does not preclude a role in HC function for WHRN-M, which contains one and part of another PDZ domain, the stereocilia phenotype in the *Whrn*$^{wi/wi}$ mouse manifests across all HCs despite the predicted expression of WHRN-M in this line.

That *Whrn*$^{tm1b/tm1b}$ mutants had normal IHC morphology, tip links, and a lack of profound deafness, suggests their IHCs are at least partly functional. Conversely, abnormal OHC morphology, poor frequency tuning, and raised DPOAE thresholds (40 dB higher than littermate controls) suggest impaired OHC function. Indeed, the electrophysiological responses of *Whrn*$^{tm1b/tm1b}$ mutants are very similar to those reported for the prestin mutant (Cheatham et al., 2004), which are believed to have abnormal OHC but normal IHC function.

WHRN has previously been described as localizing to the stereocilia tip and base region (Mathur et al., 2015b; Michalski et al., 2007; Zou et al., 2014). Using immunofluorescence at subdiffraction limit resolution afforded by SIM, we found that while WHRN-S localizes to stereocilia tips (consistent with previous reports), WHRN-L localizes toward the middle of the stereocilia rather than the base. This localization leads us to propose that WHRN-L may have a role in scaffolding inter-stereociliary links such as lateral links or horizontal top-connectors, that join adjacent stereocilia within and between rows (Hackney and Furness, 2013). In such a scenario, the lack of WHRN-L may result in weakened scaffolding of inter-stereociliary links and compromised OHC function. We detected some lateral links between adjacent stereocilia in P28 *Whrn*$^{tm1b/tm1b}$ mutants (Figure S3), although this does not preclude a potential role for WHRN-L as a scaffold.

In the *Eps8* null mutant, WHRN-S does not localize properly to stereocilia tips but WHRN-L localization is unchanged, suggesting that WHRN-S requires EPS8 for normal stereocilia tip localization. WHRN-L localization is diminished in *Eps8* null IHCs, suggesting that WHRN-L and EPS8 interactions may also be hair cell-specific. Similarly, it is interesting that the absence of WHRN-L results in shorter stereocilia only in certain vestibular hair cells. Together these observations, summarized in Figure 5I, suggest that there are multiple pathways or networks, comprising distinct molecular players, for stereocilia length regulation in different cell types. In line with isoform-specific functions described in this study, two isoforms of MYO15 were recently shown to selectively traffic to different stereocilia rows, where they have independent functions (Fang et al., 2015). Possible mechanisms underlying the differential targeting of WHRN isoforms include: (1) specific combinations of MYO15/WHRN isoforms form the basis for targeted trafficking of the different complexes within stereocilia; (2) WHRN-L, with its additional PDZ domains, is sterically restricted from being trafficked to stereocilia tips; and (3) WHRN-S requires active transport by a molecular motor to target stereocilia tips, while passive transport may be sufficient for the more proximal localization of WHRN-L. While mechanistic details remain to be elucidated, the data thus far paint a picture of the WHRN/EPS8/MYO15 complex as having multiple roles regulated by its constituent isoforms. It is becoming increasingly clear, from these studies and others, that many stereocilia-associated genes have multiple protein-coding products with not only distinct, but also very different functions. Additionally, to obtain a holistic picture of the molecular basis underlying stereocilia bundle formation, maintenance, and function, expression profiles of even well-known stereocilia-associated proteins must be considered across HC-type and their localizations carefully assessed within the stereocilia bundle.

EXPERIMENTAL PROCEDURES

Production and Genotyping of *Whrn* Mutants

The *Whrn*^{tm1b} allele was generated by mating *Whrn*$^{tm1a(KOMP)Wtsi}$ mice, produced at the Wellcome Trust Sanger Institute, to *Hprt*$^{Tg(CMV-Cre)Brd}$ mice that express Cre recombinase widely. The *Whrn*$^{wi/wi}$ allele originated on an undefined genetic background and has since been maintained within a closed colony for over 30 years. Details are in the Supplemental Experimental Procedures.

RNA Extraction and RT-PCR

RNA was extracted from cochlear and vestibular organs from mouse inner ears using QIAshredder columns (QIAGEN, cat. no. 79654) and the RNeasy mini kit (QIAGEN, cat. no. 74104), or the Lexogen SPLIT kit (Lexogen, cat. no. 008.48). RNA concentration was measured using a Nanodrop spectrophotometer (ND-8000). cDNA was made using Superscript II Reverse Transcriptase (Invitrogen, cat. no. 11904-018) before Sanger sequencing. Details in the Supplemental Experimental Procedures.

Immunofluorescence Microscopy

Organ of Corti tissue was labeled using antibodies against mouse WHRN (PB384 and PB595) and mouse EPS8 and viewed in a Nikon inverted fluorescence microscope, outfitted with a spinning disk confocal scan head or an N-SIM Super Resolution System, 100× Apo TIRF 1.49 NA objective, and a CMOS camera. NIS-Elements imaging software was utilized for image acquisition and reconstruction. Details are in the Supplemental Experimental Procedures.

Statistical Methods

Means and SDs were calculated using Microsoft Excel.

ACCESSION NUMBERS

The accession numbers for the isoforms reported in this paper are GenBank: AY739121.1 and GenBank: NM_001008795.1.

SUPPLEMENTAL INFORMATION

Supplemental Information includes Supplemental Experimental Procedures, four figures, and two tables and can be found with this article online at http://dx.doi.org/10.1016/j.celrep.2016.03.081.

AUTHOR CONTRIBUTIONS

S.E. designed experiments and collected and analyzed scanning electron microscopy and immunolocalization data; M.A.L. carried out the molecular analysis; N.I., M.A.L., and J.C.P. carried out the ABR analysis; M.J.C.R. carried out the round window recordings; J.C.P. produced the tm1b allele; and R.C. and B.K. labeled and imaged the *Eps8* mutant sample and validated antibodies in COS7 cells. All authors analyzed the results. K.P.S. led the project and S.E. wrote the paper and generated the figures. All authors contributed to the final version.

ACKNOWLEDGMENTS

We thank the Wellcome Trust Sanger Institute's Mouse Genetics Project for generating the $Whrn^{tm1a}$ mouse allele, Jonathan Gale for the FM1-43 dye, Victoria Rook for genotyping, colony management, and LacZ staining, Lorenzo Preite for genotyping, Roz Lacey for contact righting tests, Selina Pearson for initial ABR screening of the tm1a allele, and Allan Bradley and Haydn Prosser for providing the $Hprt^{Tg(CMV\text{-}Cre)Brd}$ mouse line. The work was supported by the Wellcome Trust (100669 and 098051 to K.P.S.), the MRC (K.P.S. and M.J.C.R.), Action on Hearing Loss (S.E. and K.P.S.) and by NIH intramural research funds Z01-DC000002 (to B.K.).

Received: August 19, 2015
Revised: February 18, 2016
Accepted: March 22, 2016
Published: April 21, 2016

REFERENCES

Aller, E., Jaijo, T., van Wijk, E., Ebermann, I., Kersten, F., García-García, G., Voesenek, K., Aparisi, M.J., Hoefsloot, L., Cremers, C., et al. (2010). Sequence variants of the DFNB31 gene among Usher syndrome patients of diverse origin. Mol. Vis. *16*, 495–500.

Aranyosi, A.J., and Freeman, D.M. (2004). Sound-induced motions of individual cochlear hair bundles. Biophys. J. *87*, 3536–3546.

Audo, I., Bujakowska, K., Mohand-Saïd, S., Tronche, S., Lancelot, M.E., Antonio, A., Germain, A., Lonjou, C., Carpentier, W., Sahel, J.A., et al. (2011). A novel DFNB31 mutation associated with Usher type 2 syndrome showing variable degrees of auditory loss in a consanguineous Portuguese family. Mol. Vis. *17*, 1598–1606.

Besnard, T., Vaché, C., Baux, D., Larrieu, L., Abadie, C., Blanchet, C., Odent, S., Blanchet, P., Calvas, P., Hamel, C., et al. (2012). Non-USH2A mutations in USH2 patients. Hum. Mutat. *33*, 504–510.

Cheatham, M.A., Huynh, K.H., Gao, J., Zuo, J., and Dallos, P. (2004). Cochlear function in Prestin knockout mice. J. Physiol. *560*, 821–830.

Dallos, P., Schoeny, Z.G., and Cheatham, M.A. (1972). Cochlear summating potentials. Descriptive aspects. Acta Otolaryngol. Suppl. *302*, 1–46.

Delprat, B., Michel, V., Goodyear, R., Yamasaki, Y., Michalski, N., El-Amraoui, A., Perfettini, I., Legrain, P., Richardson, G., Hardelin, J.P., and Petit, C. (2005). Myosin XVa and whirlin, two deafness gene products required for hair bundle growth, are located at the stereocilia tips and interact directly. Hum. Mol. Genet. *14*, 401–410.

Dror, A.A., and Avraham, K.B. (2009). Hearing loss: mechanisms revealed by genetics and cell biology. Annu. Rev. Genet. *43*, 411–437.

Ebermann, I., Scholl, H.P., Charbel Issa, P., Becirovic, E., Lamprecht, J., Jurklies, B., Millán, J.M., Aller, E., Mitter, D., and Bolz, H. (2007). A novel gene for Usher syndrome type 2: mutations in the long isoform of whirlin are associated with retinitis pigmentosa and sensorineural hearing loss. Hum. Genet. *121*, 203–211.

Fang, Q., Indzhykulian, A.A., Mustapha, M., Riordan, G.P., Dolan, D.F., Friedman, T.B., Belyantseva, I.A., Frolenkov, G.I., Camper, S.A., and Bird, J.E. (2015). The 133-kDa N-terminal domain enables myosin 15 to maintain mechanotransducing stereocilia and is essential for hearing. eLife *4*, e08627.

Gale, J.E., Marcotti, W., Kennedy, H.J., Kros, C.J., and Richardson, G.P. (2001). FM1-43 dye behaves as a permeant blocker of the hair-cell mechanotransducer channel. J. Neurosci. *21*, 7013–7025.

Hackney, C.M., and Furness, D.N. (2013). The composition and role of cross links in mechanoelectrical transduction in vertebrate sensory hair cells. J. Cell Sci. *126*, 1721–1731.

Harvey, D., and Steel, K.P. (1992). The development and interpretation of the summating potential response. Hear. Res. *61*, 137–146.

Holme, R.H., Kiernan, B.W., Brown, S.D., and Steel, K.P. (2002). Elongation of hair cell stereocilia is defective in the mouse mutant whirler. J. Comp. Neurol. *450*, 94–102.

Manor, U., Disanza, A., Grati, M., Andrade, L., Lin, H., Di Fiore, P.P., Scita, G., and Kachar, B. (2011). Regulation of stereocilia length by myosin XVa and whirlin depends on the actin-regulatory protein Eps8. Curr. Biol. *21*, 167–172.

Mathur, P.D., Vijayakumar, S., Vashist, D., Jones, S.M., Jones, T.A., and Yang, J. (2015a). A study of whirlin isoforms in the mouse vestibular system suggests potential vestibular dysfunction in DFNB31-deficient patients. Hum. Mol. Genet. *24*, 7017–7030.

Mathur, P.D., Zou, J., Zheng, T., Almishaal, A., Wang, Y., Chen, Q., Wang, L., Vashist, D., Brown, S., Park, A., and Yang, J. (2015b). Distinct expression and function of whirlin isoforms in the inner ear and retina: an insight into pathogenesis of USH2D and DFNB31. Hum. Mol. Genet. *24*, 6213–6228.

Mburu, P., Mustapha, M., Varela, A., Weil, D., El-Amraoui, A., Holme, R.H., Rump, A., Hardisty, R.E., Blanchard, S., Coimbra, R.S., et al. (2003). Defects in whirlin, a PDZ domain molecule involved in stereocilia elongation, cause deafness in the whirler mouse and families with DFNB31. Nat. Genet. *34*, 421–428.

Michalski, N., Michel, V., Bahloul, A., Lefèvre, G., Barral, J., Yagi, H., Chardenoux, S., Weil, D., Martin, P., Hardelin, J.P., et al. (2007). Molecular characterization of the ankle-link complex in cochlear hair cells and its role in the hair bundle functioning. J. Neurosci. *27*, 6478–6488.

Mogensen, M.M., Rzadzinska, A., and Steel, K.P. (2007). The deaf mouse mutant whirler suggests a role for whirlin in actin filament dynamics and stereocilia development. Cell Motil. Cytoskeleton *64*, 496–508.

Mustapha, M., Chouery, E., Chardenoux, S., Naboulsi, M., Paronnaud, J., Lemainque, A., Mégarbané, A., Loiselet, J., Weil, D., Lathrop, M., and Petit, C. (2002). DFNB31, a recessive form of sensorineural hearing loss, maps to chromosome 9q32-34. Eur. J. Hum. Genet. *10*, 210–212.

Offenhäuser, N., Castelletti, D., Mapelli, L., Soppo, B.E., Regondi, M.C., Rossi, P., D'Angelo, E., Frassoni, C., Amadeo, A., Tocchetti, A., et al. (2006). Increased ethanol resistance and consumption in Eps8 knockout mice correlates with altered actin dynamics. Cell *127*, 213–226.

Skarnes, W.C., Rosen, B., West, A.P., Koutsourakis, M., Bushell, W., Iyer, V., Mujica, A.O., Thomas, M., Harrow, J., Cox, T., et al. (2011). A conditional knockout resource for the genome-wide study of mouse gene function. Nature *474*, 337–342.

Stepanyan, R., Belyantseva, I.A., Griffith, A.J., Friedman, T.B., and Frolenkov, G.I. (2006). Auditory mechanotransduction in the absence of functional myosin-XVa. J. Physiol. *576*, 801–808.

Tlili, A., Charfedine, I., Lahmar, I., Benzina, Z., Mohamed, B.A., Weil, D., Idriss, N., Drira, M., Masmoudi, S., and Ayadi, H. (2005). Identification of a novel frameshift mutation in the DFNB31/WHRN gene in a Tunisian consanguineous family with hereditary non-syndromic recessive hearing loss. Hum. Mutat. *25*, 503.

Yang, J., Liu, X., Zhao, Y., Adamian, M., Pawlyk, B., Sun, X., McMillan, D.R., Liberman, M.C., and Li, T. (2010). Ablation of whirlin long isoform disrupts the USH2 protein complex and causes vision and hearing loss. PLoS Genet. *6*, e1000955.

Zou, J., Zheng, T., Ren, C., Askew, C., Liu, X.P., Pan, B., Holt, J.R., Wang, Y., and Yang, J. (2014). Deletion of PDZD7 disrupts the Usher syndrome type 2 protein complex in cochlear hair cells and causes hearing loss in mice. Hum. Mol. Genet. *23*, 2374–2390.

Cell Reports, Volume *15*

Supplemental Information

Alternative Splice Forms Influence Functions of Whirlin in Mechanosensory Hair Cell Stereocilia

Seham Ebrahim, Neil J. Ingham, Morag A. Lewis, Michael J.C. Rogers, Runjia Cui, Bechara Kachar, Johanna C. Pass, and Karen P. Steel

Figure S1

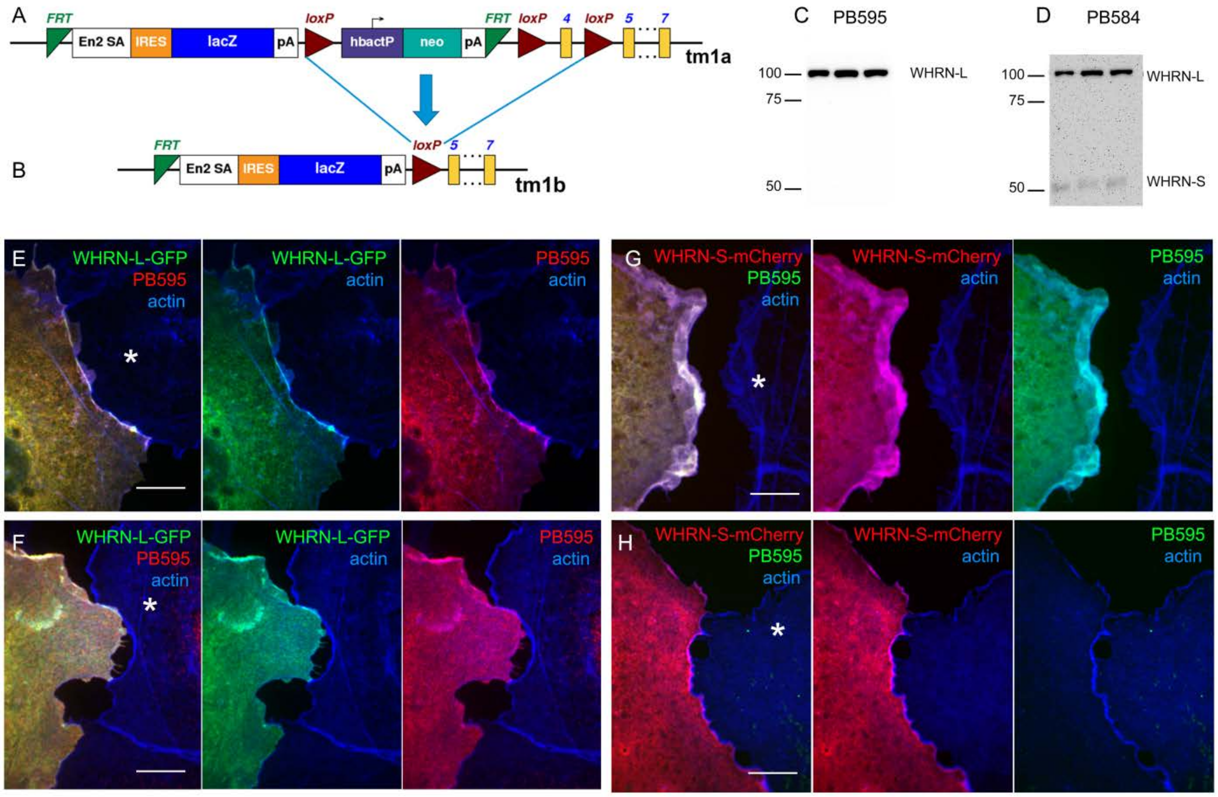

Figure S1. - Generation of *Whrn*^{tm1a} and *Whrn*^{tm1b} alleles, and validation of anti-WHRN antibodies. (A and B) Generation of *Whrn*^{tm1a} and *Whrn*^{tm1b} alleles. (A) Diagram representing the *Whrn*$^{tm1a(KOMP)Wtsi}$ allele. Exons are indicated by yellow rectangles. The large selection cassette is surrounded by FRT sites (green triangles) and LoxP sites (red triangles) flanking exon 4 allow excision by exposure to Cre recombinase. Transcription of the tm1a allele is inhibited by the large extraneous DNA insertion, acting in a similar way to a gene-trap allele. (B) The tm1b allele is derived from the tm1a allele by crossing with *Hprt*$^{Tg(CMV-Cre)Brd}$ mice, which express Cre recombinase, leading to recombination between two LoxP sites, thereby deleting exon 4 and part of the inserted cassette. (C and D) Western blots from retina and inner ear tissue, combined, from three *Whrn*$^{+/tm1b}$ mice probed with PB595 (C) and PB584 after stripping (D). (E - H) Validation of anti-WHRN antibodies. COS7 cells were transfected with WHRN-L-GFP (green) and labeled with PB584 (E) or PB595 (F), an anti-rabbit secondary antibody conjugated to Alexa Fluor 568 (red), and phalloidin to mark actin (blue). Colocalization (yellow signal) between WHRN-L-GFP and PB584 (E) or PB595 (F) in the transfected cell confirms antibody specificity. Lack of red signal in the untransfected cells (asterisks) confirms antibody selectivity. COS7 cells were also transfected with WHRN-S-mCherry (red) and labeled with PB584 (G) or PB595 (H), an anti-rabbit secondary antibody conjugated to Alexa Fluor 488 (green), and phalloidin (blue). Colocalization (yellow signal) between WHRN-S-mCherry and PB584 (G) in the transfected cell confirms antibody specificity for WHRN-S. No green signal was observed in transfected cells labeled with PB595 (H), confirming that PB595 does not label WHRN-S. Scale bar = 10 µm. **Related to Figure 1 and 4**

Figure S2

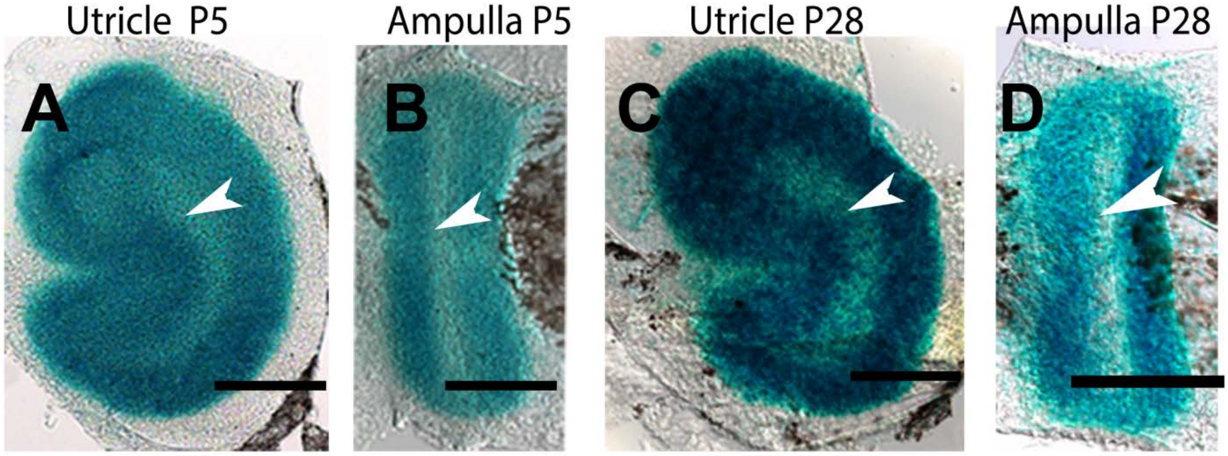

Figure S2. LacZ staining in vestibular sensory epithelia from *Whrn*$^{tm1b/tm1b}$ mice. At both P5 (A and B) and P28 (C and D) the label is weaker in the striolar region of the utricle and the central zone of the ampullae (arrowheads). Scale bars = 500 µm. **Related to Figure 2.**

Figure S3

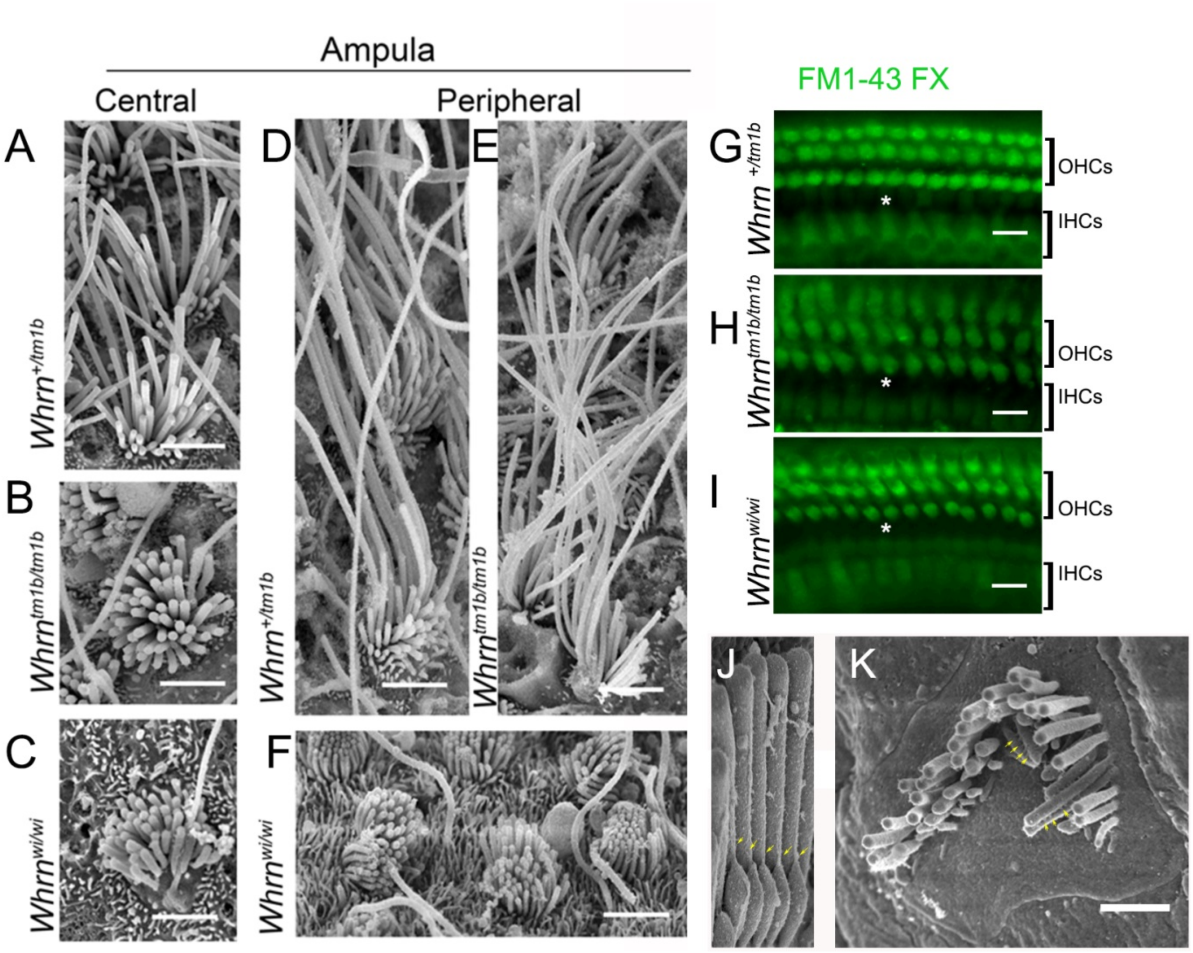

Figure S3. Cristae ampularis bundle morphology, and FM1-43 uptake in ***Whrn*** $^{tm1b/tm1b}$ **and** ***Whrn*** $^{wi/wi}$ **mutants.** Examples of stereocilia bundle morphology in the central zone in *Whrn* $^{+/tm1b}$ (A), *Whrn* $^{tm1b/tm1b}$ (B) and *Whrn* $^{wi/wi}$ (C) mice at P21. Examples of peripheral zone bundles in *Whrn* $^{+/tm1b}$ (D), *Whrn* $^{tm1b/tm1b}$ (E) and *Whrn* $^{wi/wi}$ (F) mice. (G-I) FM1-43 dye (green) applied to the organ of Corti showed loading in both IHCs and OHCs of *Whrn* $^{+/tm1b}$ mice (G) as well as *Whrn* $^{tm1b/tm1b}$ (H) and *Whrn* $^{wi/wi}$ (I) mutants. Support cells do not show any dye uptake (white asterisks). (J and K) Some inter-stereocilia links are present in the *Whrn* $^{tm1b/tm1b}$ mutant. SEM of IHC stereocilia from *Whrn* $^{tm1b/tm1b}$ mutants (P28) showing the presence of tip links (J, yellow arrows). SEM of OHC stereocilia from *Whrn* $^{tm1b/tm1b}$ mutants (P28) showing the presence of lateral links between adjacent stereocilia (K, yellow arrows). Scale bars A-F= 2 µm; G-1 = 10 µm; K = 1 µm. **Related to Figure 3.**

Figure S4

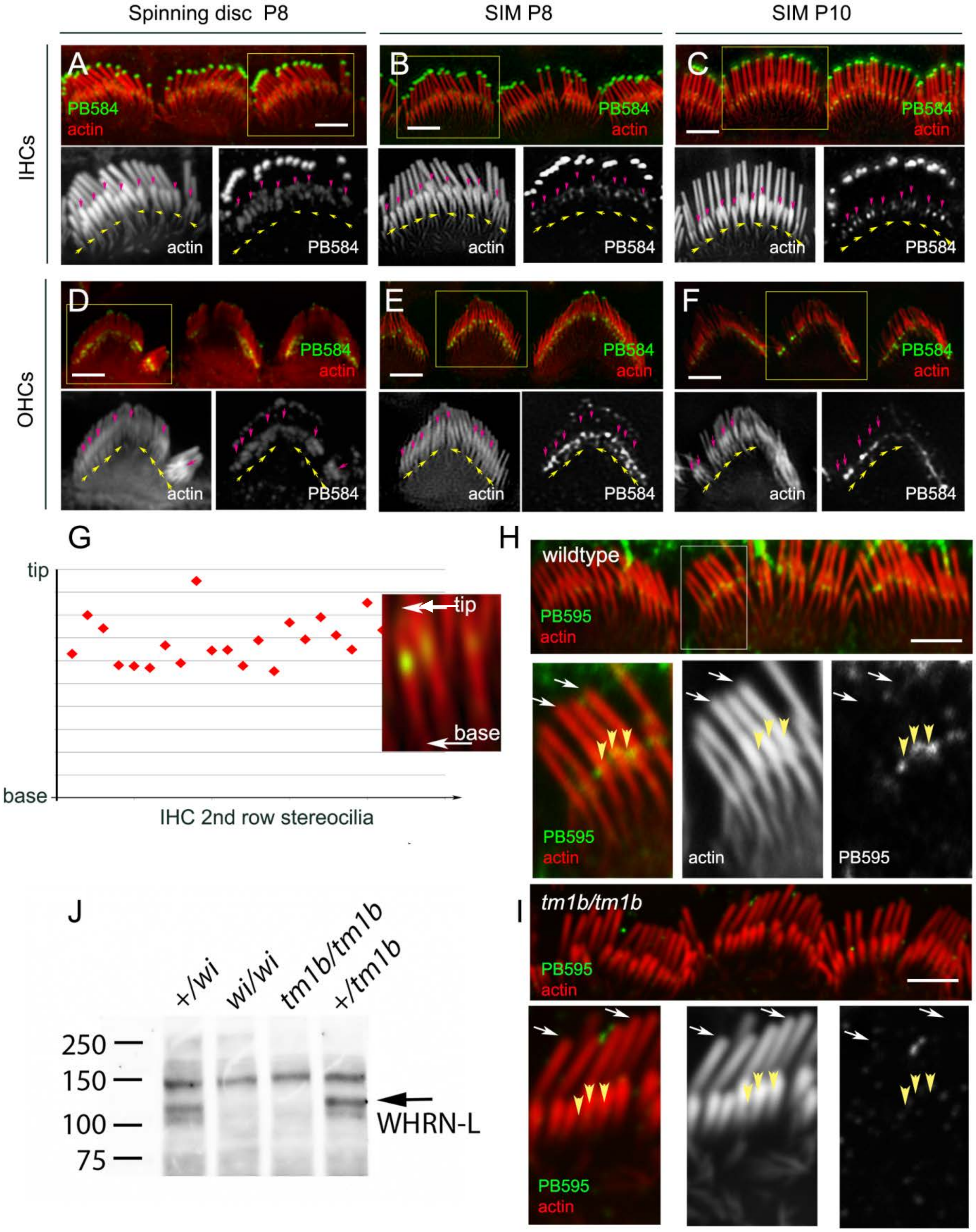

Spinning disc P8
SIM P8
SIM P10
IHCs
OHCs
A
B
C
D
E
F
PB584
actin
G
tip
base
IHC 2nd row stereocilia
H
wildtype
PB595
actin
I
tm1b/tm1b
J
+/wi
wi/wi
tm1b/tm1b
+/tm1b
250
150
100
75
WHRN-L

Figure S4. WHRN-L localization in IHC and OHC stereocilia. (A-C) IHC stereocilia (red) labeled with PB584 (green) to detect both WHRN-L and WHRN-S, and imaged using conventional spinning disc microscopy at P8 (A) and super-resolution SIM at P8 (B) and P10 (C). Pink arrowheads point to tips of second row stereocilia, and yellow arrowheads point to stereocilia bases. (D-F) OHC stereocilia (red) labeled with PB584 (green) to detect both WHRN-L and WHRN-S, and imaged using conventional spinning disc microscopy at P8 (D) and SIM at P8 (E) and P10 (F). Pink arrowheads point to tips of second row stereocilia, and yellow arrowheads point to stereocilia bases. (G) Plot showing the localization of WHRN-L (red dots) relative to the tip and base of second row stereocilia. Inset- magnified view of second row stereocilia with WHRN-L labeled (green) and tip and base highlighted (white arrows). (H and I) IHC stereocilia (red) from wildtype (H) and tm1b/tm1b (I) mice at P10 labeled with PB595, an antibody specific to WHRN-L (green). White arrows point to tips of tallest row of stereocilia. Arrowheads points to region of WHRN-L localization, proximal to the tips of the second row of stereocilia. (J) Western blot of brain tissue from +/wi, wi/wi, +/tm1b and tm1b/tm1b mice probed with PB595 (against WHRN-L). Scale bars= 2 µm. **Related to Figure 4.**

Table S1- WHRN isoforms with corresponding Ensembl and GenBank Accession Numbers

Isoform	Ensembl Transcript ID	Genbank IDs
WHRN-L	ENSMUST00000084510	NM_001008791
WHRN-L	ENSMUST00000107393	NM_001008792
WHRN-L	ENSMUST00000063650	NM_028640
WHRN-L	ENSMUST00000102867	NM_001008793
WHRN-M	ENSMUST00000063672	AK157955.1
WHRN-S	ENSMUST00000095038	NM_001008794
WHRN-S	ENSMUST00000119294	NM_001008795.1
WHRN-S	ENSMUST00000095037	NM_001008796
WHRN-N	ENSMUST00000133425	NM_001276371
WHRN-N	ENSMUST00000144965	BM940881.1
WHRN-S	ENSMUST00000155058	AY739121.1

Table S2- Primers used to amplify *Whrn* protein-coding transcripts

Exon	Primer name	Sequence
ENSMUSE00000603856, ENSMUSE00001218627	1F	CTGCAGCCCCAGGATTTTAC
ENSMUSE00000662852	2F	ACGATAAATCTCTAGCCCGGG
ENSMUSE00001218627	3F	AGTACCCTCCACCTCCTGC
ENSMUSE00001236620	4F	CACACAGAAGAAGGGACAAGC
ENSMUSE00000789315	f	GTAGGAGATGCGAGCACTTTGTACGC
ENSMUSE00001278579	5F	CGCCACCATGATGTACTACC
ENSMUSE00000747140	1R	GTGTGAGACCTTCTTGCTGG
ENSMUSE00000776975	2R	GACAGACTCTCCCATGGCC
ENSMUSE00001268183	3R	GTCTTGGGGTGAGATGATGC
ENSMUSE00000222478	5R	TCTTAGATGCCCACTGTCCC
ENSMUSE00001245215	d	GTTAACCAGGGCCAGGTGGGTGTC
ENSMUSE00001245215	6R	GTCATGACCTTGAGCCAGG
ENSMUSE00001271849	7R	CTGCACTTTTCCTCACACGG
ENSMUSE00001295720	8R	TGCCTTCCTTCCCTTCACC

Isoform	Specific primer sets
WHRN-N	2F/1R, 3F/2R
WHRN-M	5F/5R
WHRN-S	f/d, 1F/3R
WHRN-L	3F/6R, 3F/7R, 4F/6R, 4F/7R, 4F/8R

Supplemental Experimental Procedures

Ethics statement

Mouse studies were carried out in accordance with UK Home Office regulations and the UK Animals (Scientific Procedures) Act of 1986 under Home Office licences, and the study was approved by both the Wellcome Trust Sanger Institute and the King's College London Ethical Review Committees. Mice were culled using methods approved under these licences to minimize any possibility of suffering. *Eps8* null mice experiments were conducted in accordance with animal protocols approved by the NIH Animal Care and Use Committee (Protocol #1264).

Production and genotyping of *Whrn* mutants

Whrn$^{tm1a(KOMP)Wtsi}$ mice were produced at the Wellcome Trust Sanger Institute and carry a knockout-first conditional-ready allele [1, 2]. The mice were maintained in individually-ventilated cages at a standard temperature and humidity and in specific pathogen-free conditions on a C57BL/6N genetic background. For genotyping, DNA was extracted from ear-clips and used for short range PCR. For the tm1a allele, wild type primers were: forward GCTCATTTGCCCATTGTTTTC, reverse CTGAAGTCCCCCACCAGAAG, product size of 505bp. The mutant allele shares the forward primer with wild type and the reverse primer TCGTGGTATCGTTATGCGCC to produce a 196bp product. The wild type reaction spans exons 3-4 and fails in the presence of the cassette as the sequence is too large to be amplified by short range PCR. *Whrn*$^{tm1a/tm1}$a mutants showed ABR response thresholds very similar to those of *Whrn*$^{tm1b/tm1b}$ (data not shown), so the latter allele was used for the remaining experiments.

The *Whrn*^{tm1b} allele was generated by mating mice carrying the tm1a allele to *Hprt*$^{Tg(CMV\text{-}Cre)Brd}$ mice which express Cre recombinase widely, resulting in recombination between LoxP sites in the *Whrn*^{tm1a} allele. The tm1b allele was genotyped using the same primers used for the tm1a allele. The CMV-Cre allele was bred out of the colony before phenotypic characterization. The primers used to detect the wild type sequence at the *Hprt* locus were: forward CTTTCCTCATGCCCCAAAATCTTAC and reverse ATGTAATCCAGCAGGTCAGCAAGA to produce a 311 bp product. For the mutant *Hprt*$^{Tg(CMV\text{-}Cre)Brd}$ allele , the forward primer is shared with the wild type and the reverse primer was GCTATCAGGACATAGCGTTGGCTAC, giving a 700 bp product. Primers for LoxP (forward ATCCGGGGGTACCGCGTCGAG and reverse ACTGATGGCGAGCTCAGACC) and tm1b (forward CGGTCGCTACCATTACCAGT, reverse is shared with LoxP) were used to confirm recombination and conversion into a tm1b allele. After complete conversion to a tm1b allele the LoxP reaction fails as only one LoxP site remains. In the tm1a allele the LoxP PCR product is between 800bp and 1kb and is reduced to 130bp if only the critical exon is removed (see Figure 1A, B). When recombination removes only the *neo* gene and not exon 4, the tm1b reaction produces a 1kb product. Following full conversion to a tm1b allele, where both the *neo* and exon 4 are removed, the tm1b reaction product size is 380bp.

The *Whrn*$^{wi/wi}$ allele originated on an undefined genetic background and has since been maintained within a closed colony for over 30 years. Round window recordings were carried out using mice maintained in a conventional facility at a standard temperature and humidity using homozygous mutants and heterozygous littermate controls. All other experiments were carried out after rederivation into specific pathogen-free conditions in individually-ventilated cages. Primers for genotyping were: wild type forward ATGAAAGTGCCACGGAGATG and wild type reverse TGCACTCTGTCCCTGACTCC, and the mutant reverse CCAGAGCTGGACAACCATAC which shares the wild type forward primer, giving a wild type product size of 476bp and a mutant allele product size of 678bp. The wild type reverse primer is within the deleted region, so a product should not be generated for the mutant allele. The mutant reverse is on the other side of the deletion and should not generate a product for extension times below 45 s.

RNA extraction and RT-PCR

Cochlear and vestibular organs from mouse inner ears were dissected in RNAlater (Ambion, cat. no. AM7024) or phosphate buffered saline (PBS), pH 7.4 and stored in RNAlater. RNA was extracted using QIAshredder columns (QIAgen, cat. no. 79654) and the RNeasy mini kit (QIAgen, cat. no. 74104), or the Lexogen SPLIT kit (Lexogen,

cat. no, 008.48), following the manufacturer's instructions. RNA concentration was measured using a Nanodrop spectrophotometer (ND-8000). cDNA was made using Superscript II Reverse Transcriptase (Invitrogen, cat. no. 11904-018) after treatment with DNAse 1 (Sigma, cat.no: AMP-D1). Primer sequences are in Table S2. Sanger sequencing was carried out by Source BioScience and analysed using Gap4 [3]. We sequenced cDNA from 6 $Whrn^{tm1b/tm1b}$ and 2 $Whrn^{+/tm1b}$ mice, 5 $Whrn^{wi/wi}$ and 1 $Whrn^{+/wi}$ mice, 3 $Whrn^{+/+}$ mice and 2 unrelated C57BL6/N wildtype mice as additional controls.

Western Blots

Retinas, inner ear sensory epithelia or brain tissue from each mouse was homogenized in 300 µl/100 mg tissue RadioImmune Precipitation Assay (RIPA) buffer, sonicated and boiled with Laemmli buffer for 10 min. The samples were separated by SDS polyacrylamide gel electrophoresis and transferred to a polyvinylidene fluoride (PVDF) membrane. The resulting PVDF membrane was sequentially subjected to blocking for 1 h, primary antibody (PB595) incubation overnight at 4°C and secondary antibody incubation for 1 h. The membrane was stripped prior to labelling with PB584. The protein bands were detected using ECL chemiluminescent substrate (Thermo Scientific Pierce).

Cochlear electrophysiology.

Detailed descriptions of these methods are described elsewhere [4]. In brief, mice were anaesthetized with urethane, 20 $mg.g^{-1}$ intraperitoneal injections, the middle ear was opened and a Teflon-coated silver wire-recording electrode was placed on the round window, the ground at the vertex and reference on muscle behind the ear. A closed, calibrated sound delivery system was placed in the external ear canal. Cochlear microphonics were recorded using continuous tones stepped in frequency and intensity, recorded using a Brookdeal Lock-in amplifier. Summating potentials and compound action potentials were recorded using shaped tonebursts with rise/fall times of 1 ms, duration 15 ms, repetition rate 8.6 s^{-1}. A total of 28 whirler homozygotes plus 28 heterozygous littermate controls were analyzed with at least 6 in each group at ages P13, P15, P17 and P20.

Auditory Brainstem Electrophysiology (ABR).

Detailed descriptions of these methods are described in [5]. Mice were anaesthetised using an intraperitoneal injection of either urethane (2mg/g) or ketamine hydrochloride (100 $mg.kg^{-1}$, Ketaset, Fort Dodge Animal Health) and xylazine hydrochloride (10 mg/Kg, Rompun, Bayer Animal Health). Subcutaneous needle electrodes were placed over the right bulla (ground), left bulla (reference) and vertex (active). Electroencephalographic potentials were amplified, filtered (300 Hz-3 kHz) and averaged to produce the ABR. ABRs were recorded for calibrated tones (5ms duration, 1ms rise / fall time, 6-30 kHz) and clicks (10 µs duration) presented freefield at either 21.3 s^{-1} or 42.6 s^{-1} at a range of stimulus levels to determine threshold (dB SPL). Numbers of mice used: $Whrn^{wi/wi}$ P20 n=20, P56 n=2, P98 n=5; $Whrn^{+/wi}$ P20 n=16, P56 n=7, P98 n=5; $Whrn^{+/+}$ P20 n=4, P56 n=7. $Whrn^{tm1a/tm1a}$ P98 n=4, $Whrn^{+/tm1a}$ n=4, $Whrn^{+/+}$n=4. $Whrn^{tm1b/tm1b}$P28-35 n=8, P98 n=14; $Whrn^{+/tm1b}$ P28-35 n=4, P98 n=15; $Whrn^{+/+}$P28-35 n=9; P98 n=6.

ABR recordings were used to measure frequency tuning curves with a modified forward masking paradigm. A probe tone (12 kHz, 5ms duration, 1ms rise/fall time, presented at threshold +20dB) was presented with a 4ms gap after a masker tone of variable frequency, from 6 – 19.2 kHz in 9 steps, 10ms duration, with a 1ms rise/fall time, presented at levels ranging from 0-90dB SPL in 10dB steps. Masked threshold of the 12kHz probe tone was estimated for each masker frequency as the masker level that resulted in a 3dB (50% magnitude) reduction in ABR wave 1 amplitude modified from [6], and plotted as a function of masker frequency to produce an ABR frequency tuning curve for each mouse. Six $Whrn^{tm1b/tm1b}$ and 6 $Whrn^{+/tm1b}$ mice aged 14 weeks were used.

Distortion Product Otoacoustic Emission (DPOAE) measurements.

We measured distortion product otoacoustic emissions in $Whrn^{tm1b}$ mice aged 14 weeks old, anaesthetised with Urethane, 0.1ml/10g bodyweight of a 20% solution, ip. We used Tucker Davis Technologies (TDT) BioSigRZ software, running on a TDT RZ6 auditory processor, driving a pair of TDT EC1 electrostatic transducers, with signals being recorded via an Etymotic ER-10B+ low noise DPOAE microphone. Stimuli were presented and signals recorded via a closed-field acoustic system sealed into the auditory meatus of the mouse. Stimulus tones

were presented in an F2:F1 ratio of 1.2. F2 tones were presented at 6, 12, 18, 24 & 30 kHz. F1 was presented at levels from 0-75dB in 5 dB steps. F2 was presented at 10 dB below the level of F1. The magnitude of the 2F1-F2 DPOAE component was extracted from a fast Fourier transform of the recorded microphone signal and plotted as a function of F2 level. For each F2 level, the 20 spectral line magnitudes surrounding the 2F1-F2 frequency were averaged to form a noise floor for each measurement. DPOAE threshold was defined as the lowest stimulus level where the emission magnitude exceeded 2 standard deviations above the mean noise floor. n=5 $Whrn^{tm1b/tm1b}$, 3 $Whrn^{+/tm1b}$.

Contact righting reflex

Adult mice were placed in a large glass petri dish, the lid positioned allowing the mouse to move but with contact of its feet and back with the glass, and the dish was turned over. The response of the mouse was observed and timed. Mice with normal balance turn over immediately, but mice with reduced balance function take longer to turn, presumably because the lack of vestibular input slows their ability to detect their position in relation to gravity. n=4 $Whrn^{tm1a/tm1a}$, 4 $Whrn^{+/tm1a}$, 4 +/+; 4 $Whrn^{tm1b/tm1b}$, 4 $Whrn^{+/tm1b}$, *4 +/+; 4* $Whrn^{wi/wi}$, *4* $Whrn^{+/wi}$

LacZ expression analysis

Inner ears (n= 1 heterozygote and 2 homozygotes at P5 and P28) were dissected out and fixed in fresh 4% paraformaldehyde for 45 minutes at 4^0C with rotation, decalcified in 10% EDTA before a detergent wash (2 mM $MgCl_2$; 0.02 % NP-40; 0.01 % sodium deoxycholate; in 0.1 M sodium phosphate buffer, pH 7.3 in PBS) for 30 min at room temperature. X-gal (Promega; cat.no. V394A) was added 1:50 to 500 µl of pre-warmed staining solution (5 mM $K_3Fe(CN)_6$; Ferrate (III) and 5 mM $K_4Fe(CN)_6$; Ferrate (II)), then ears were stained at 37°C in the dark for 1hr, washed in PBS and counterstained with Nuclear Fast Red (VWR, cat.no. 342094W). Cochlear and vestibular sensory epithelia were then microdissected, and whole mounts prepared for imaging with a Zeiss Axioskop 2.

FM1-43 uptake

For FM1-43 uptake, cochleae were processed as described in [7]. Briefly, cochleae from P10 mice (n=3) were excised and a small hole made at the apex of the bony cochlea. 1 ml of FM1-43FX (Invitrogen) at 5 µM in PBS at room temperature was perfused through the cochlear duct from oval and round windows for 30 seconds, and then washed 3X with 1 ml of PBS, followed by fixation with 4 % PFA for 20 min at room temperature. Organs of Corti were excised and mounted in ProLong Gold (Life Technologies) mounting media for imaging of the mid-apical region (60 – 70%) of the organ of Corti with a Zeiss Axioskop 2.

Immunofluorescence and stereocilia length quantification

Polyclonal antibodies against mouse WHRN (PB584 and PB595) were developed (Princeton Biomolecules and Covance) in rabbits immunized with a synthetic peptide (PB584: CDEETRKAREKERRRRLRRGA; PB595: SSLPQPHGSTLRQREDD). After affinity purification, specificity and selectivity was verified in COS7 cells transfected with WHRN-GFP (Figure S1). Rabbit polyclonal anti-EPS8 antibody (ab96144) was purchased from Abcam. Temporal bones were fixed by immersion in 4 % paraformaldehyde (PFA) in PBS; pH 7.4 for 20 min at room temperature. Sensory tissues were dissected in PBS, permeabilized with 0.5 % Triton X-100 for 30 min and blocked overnight at 4 °C with 4% bovine serum albumin in PBS. For labeling with PB595, tissue required treatment with 0.01M TRIS-EDTA (pH 9) for 45 minutes at 60 °C for antigen retrieval prior to blocking. Tissue was then incubated with primary antibody for 2 h, rinsed with PBS, stained with Alexa Fluor 488-, 568- or 405-conjugated secondary antibody (Life Technologies) for 1 h, counterstained with 0.001 U/µl Alexa Fluor 488- or 568-phalloidin (Molecular Probes), and mounted as whole sensory epithelia using Prolong Gold Antifade (Molecular Probes). Microscopy was performed using a Nikon TiE inverted fluorescence microscope, fitted with a spinning disc confocal head or an N-SIM Super Resolution System, 100x Apo TIRF 1.49 N.A. objective, and Andor Ixon camera. NIS-Elements imaging software was utilized for image acquisition. Stereocilia bundles were imaged at the mid-apical region, 60% - 70% from the basal end of the cochlea.

Stereocilia length measurements were made from confocal images, each of which contained several tens of stereocilia bundles within a single field of view and the same focal plane. Tissue was mounted by gently pressing against the coverslip to allow the bundles to virtually lay flat against the glass surface and remain within the ~500nm confocal optical section. The tallest stereocilia in each bundle was traced from base to tip, and this length was used. While the results may be a very slight underestimate of the length the relative measurements are internally consistent. Three animals of each genotype were used for the measurements.

Scanning Electron Microscopy (SEM)

The inner ear was gently flushed with ~0.3 ml of 2.5% glutaraldehyde fixative solution followed by two hours fixation at room temperature. Dissected cochlear and vestibular tissues were processed using an osmium-thiocarbohydrazide method [8], including three 1 hr incubations with 1% (w/v) OsO_4, alternated with two 1 hr incubations in 1% thiocarbohydrazide (w/v). The specimens were dehydrated, critical-point dried and viewed using a Hitachi S-4800 field emission scanning electron microscope. Stereocilia bundles were imaged at the mid-apical region, 60% - 70% from the basal end of the cochlea. N=3.

Supplemental References

1. White, J.K., et al., *Genome-wide generation and systematic phenotyping of knockout mice reveals new roles for many genes.* Cell, 2013. **154**(2): p. 452-64.
2. Skarnes, W.C., et al., *A conditional knockout resource for the genome-wide study of mouse gene function.* Nature, 2011. **474**(7351): p. 337-42.
3. Bonfield, J.K., K. Smith, and R. Staden, *A new DNA sequence assembly program.* Nucleic Acids Res, 1995. **23**(24): p. 4992-9.
4. Steel, K.P. and G.R. Bock, *Cochlear dysfunction in the jerker mouse.* Behav Neurosci, 1983. **97**(3): p. 381-91.
5. Ingham, N.J., S. Pearson, and K.P. Steel, *Using the Auditory Brainstem Response (ABR) to Determine Sensitivity of Hearing in Mutant Mice.* Curr Protoc Mouse Biol, 2011. **1**(2): p. 279-87.
6. Cheatham, M.A., et al., *Cochlear function in Prestin knockout mice.* J Physiol, 2004. **560**(Pt 3): p. 821-30.
7. Kurima, K., et al., *TMC1 and TMC2 Localize at the Site of Mechanotransduction in Mammalian Inner Ear Hair Cell Stereocilia.* Cell Rep, 2015. **12**(10): p. 1606-17.
8. Hunter-Duvar, I.M., *A technique for preparation of cochlear specimens for assessment with the scanning electron microscope.* Acta Otolaryngol Suppl, 1978. **351**: p. 3-23.
